# Differential Expression and Analysis of *TBX3* Gene in Skin Tissues of Dun Mongolian Horses with and Without Bider Markings

**DOI:** 10.3390/ani16020297

**Published:** 2026-01-18

**Authors:** Tana An, Manglai Dugarjaviin

**Affiliations:** Inner Mongolia Key Laboratory of Equine Science Research and Technology Innovation, Inner Mongolia Agricultural University, Hohhot 010018, China; 15848154479@163.com

**Keywords:** *TBX3* gene, Dun, coat color, Mongolian horse, Bider marking

## Abstract

(1) Background: The dun coat color, a wild-type phenotype in horses characterized by pigment dilution and primitive markings, is regulated by *TBX3*. This study explored the expression and localization of *TBX3* in the Bider marking (a primitive mark unique to the shoulder of horses); (2) Methods: We compared skin tissues from Bider-marked and non-Bider dun Mongolian horses. Samples were collected from the Bider area (dark-colored/light-colored shoulder), dorsal midline, and croup. Histological staining, qRT-PCR, and Western blotting were used to analyze pigment distribution and *TBX3* expression at mRNA and protein levels; (3) Results: Histology revealed asymmetric pigment deposition in hair shafts from light-colored areas of both Bider and non-Bider horses, whereas dark areas showed symmetric distribution. qRT-PCR and Western blotting showed *TBX3* expression was significantly higher in the shoulder of non-Bider horses compared to Bider horses. Conversely, Bider horses exhibited higher *TBX3* levels in all other sampled areas. Immunohistochemistry localized *TBX3* protein to the epidermis and hair follicle bulbs in both groups; (4) Conclusions: In dun Mongolian horses, *TBX3* expression differences between dark and light skin areas correlate with Bider markings. *TBX3* is implicated in this specific pigment marking, though its upstream regulation requires further study. These findings provide key insights into the mechanism behind Bider marking formation.

## Simple Summary

The significance of a horse’s coat color extends beyond mere appearance; it also serves an essential camouflage function. Some dun horses, such as the Mongolian horse, exhibit a distinctive symmetrical dark patch on their shoulders, known as the ‘Bider’ marking, which is considered a primitive trait. Our research aims to elucidate the formation of this specific marking at both histological and molecular levels, focusing on the *TBX3* gene, which is known to play a crucial role in the development of dun coloration and similar coat markings. By comparing skin samples from horses with and without this marking, we found that the *TBX3* gene is more active in light-colored skin tissues. This differential gene activity leads to variations in pigment markings in the hair. These findings enhance our understanding of the genetic mechanisms underlying this unique characteristic of the Mongolian horse, which holds significant implications for the conservation of equine genetic resources and selective breeding.

## 1. Introduction

**Figure 1:**
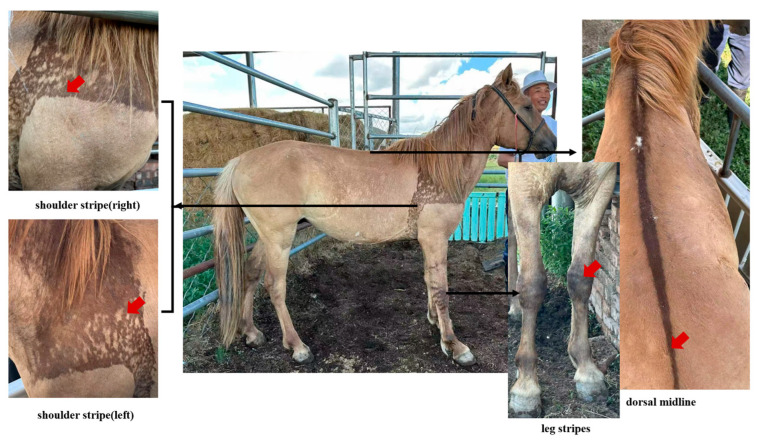
Different primitive markings formed on a dun Mongolian horse. The red arrows indicate the specific locations of the different primitive markings. The four images above are of the same horse.

The domestication history of horses and the accompanying evolution of coat color diversity provide a critical model for studying the genetic evolution of domestic horses [1,2,3,4,5]. Ancient DNA research indicates a clear chronological sequence in the evolution of domestic horse coat colors: bay is the earliest recorded coat color; black coats emerged during the Copper Age; chestnut and white spotting appeared in the Bronze Age; and diluted colors, such as palomino and silver black, began to emerge in the Iron Age [6]. Among this rich spectrum of coat colors, dun, as an ancient wild-type coat color [7], is particularly notable for its unique pigment dilution characteristics and primitive markings, such as shoulder stripe (Bider markings), dorsal midline, and leg stripes, as illustrated in Figure 1 (Supplementary Figure S1). This coloration not only facilitates the camouflage of horses in their natural environment [8] but also provides an ideal model for studying the evolution and genetic regulation of coat color in equine species [9].

The Dun phenotype exhibits a clear genetic association with the *TBX3* gene, where its dilution phenotype and the formation of primitive markings are closely linked to the asymmetric deposition of hair pigment regulated by *TBX3* [8]. Studies have demonstrated [10,11] that the dun allele is ancestral. The non-dun phenotype arises from two derived alleles: the non-dun1 (d1) allele, which is associated with primitive markings, and the non-dun2 (d2) allele, which is characterized by a derived 1.6-kb deletion and lacks primitive markings. Among the various primitive markings of Dun horses, a rare subset of individuals exhibits the Bider marking—a symmetrical, irregular black patch located on the scapular region. Research [12] indicates that the occurrence rate of the Bider marking in Mongolian horses is 0.010, with a higher prevalence observed in horses possessing the dorsal stripe primitive marking. Among 164 Przewalski’s horses, the occurrence rate of the Bider marking was found to be 0.396, which is approximately 40 times higher than that of native Mongolian horses. This marking has been documented in both male and female individuals of both horse types, indicating an autosomal inheritance marking. The Bider marking demonstrates complete dominance in the offspring produced from various hybrid combinations. Due to its specific occurrence in Przewalski’s horses and certain Mongolian horse breeds, it is considered an ideal subject for investigating the mechanisms underlying the formation of primitive markers [12]. Dun coloration encompasses three distinct types: yellow dun, grullo, and red dun. Yellow dun is derived from a bay base, whereas grullo and red dun correspond to black and chestnut bases, respectively. The Dun gene (D) is a dominant allele, which implies that any horse possessing one or two D alleles will exhibit varying degrees of coat lightening and primitive markings. There is no significant distinction between horses with one D allele (D/_) and those with two D alleles (D/D). Non-dun horses consistently exhibit genotypes of either nd1/nd1, nd1/nd2, or nd2/nd2. The different gene combinations associated with dun dilution yield diverse effects. Specifically, horses with the D/D genotype will experience a dilution of their coat color due to the dun factor, and this diluted coloration will be inherited by all their offspring. Horses with the D/nd1 or D/nd2 genotype will also display diluted coat color and primitive markings, yet they possess a 50% probability of transmitting the dun dilution to their progeny. In contrast, horses with the nd1/nd1 genotype will not exhibit dilution but may display primitive markings, and they will pass the nd1 allele to all their offspring. Similarly, horses with the nd1/nd2 genotype will not be diluted, may exhibit primitive markings, and have a 50% chance of transmitting the nd1 allele to their descendants. Horses with the nd2/nd2 genotype will neither exhibit dilution nor display primitive markings [11].

Our preliminary study focused on Dun Mongolian horses, which exhibit distinctive “Bider markings” on their shoulders. Utilizing multidisciplinary techniques, research investigated the differential expression of the *TBX3* gene across various skin regions (shoulder area, dorsal midline, and croup) of the same individual. It is important to emphasize that the primary aim of this study is not to investigate the genetic basis of the Dun phenotype [8], which is already well-established, but rather to explore how the differential expression of the *TBX3* gene elucidates the mechanisms underlying the formation of shoulder Bider markings in confirmed Dun horses. While the fundamental role of *TBX3* in pigmentation has been preliminarily established, its differential expression in specific anatomical regions—particularly the shoulders—during the formation of Bider markings remains inadequately understood. To address this critical gap, this study systematically compares the differences in hair follicle structure, pigment distribution, and *TBX3* gene expression and localization between the marked areas (light and dark-colored shoulder regions) and non-Bider-marked skin regions, utilizing the Dun Mongolian horse as a model. The findings contribute novel theoretical insights into the formation of primitive coat colors in equine species.

## 2. Materials and Methods

### 2.1. Sample Collection

**Figure 2:**
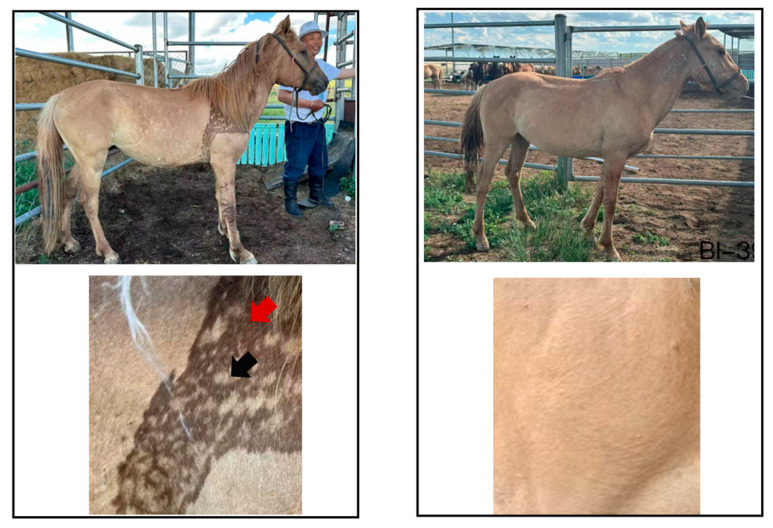
Example of sample collection from a dun Bider horse and a dun non-Bider horse. **Left**: Bider horse, with the shoulder sampling area (red arrow-dark-colored shoulder, black arrow-light-colored shoulder). **Right**: Non-Bider horse and the shoulder sampling area.

Skin tissue samples for this study were collected from the Inner Mongolia Autonomous Region of China. The samples were obtained from a population of Dun Mongolian horses, consisting of six individuals divided into two groups based on the presence of the distinctive “Bider marking.” Both groups showed other original markings, including leg stripes and back stripes. The samples included horses marked with Bider (*n* = 3) [9] and horses not marked with Bider (*n* = 3). The Bider marked group comprised two females (aged 3 and 4 years) and one male (6 years old), while the non-Bider-marked group included three males, all aged 2 years. This grouping design facilitated a comparison of potential differences in skin tissue morphology associated with the unique Bider marking trait. Each sample measured 1 × 1 cm and was obtained from three distinct anatomical sites: the shoulder, dorsal midline, and croup (Figure 2/Supplementary Figure S2). All animal experiments in this study were strictly conducted in accordance with the procedures described in Reference [9]. Equine surgeries were performed under a combined anesthesia protocol. Specifically, analgesia was achieved by intravenous administration of Detomidine (0.01–0.02 mg/kg) and Butorphanol (0.02–0.04 mg/kg) to minimize discomfort during surgery. All sample collection procedures were approved by the Animal Ethics Committee of Inner Mongolia Agricultural University [9]. Note: The Bider samples utilized in this study were sourced from [9], while the non-Bider samples were newly collected from different horses than those referenced in [9]. Both Bider and non-Bider samples exhibited the shared phenotypic characteristic of being dun in color.

### 2.2. Methods

The research methods were conducted in accordance with reference [9], encompassing paraffin sectioning, HE staining, RNA extraction, cDNA synthesis, RT-qPCR, total protein extraction, Western Blot, and immunohistochemical staining. Supplement: Xylene (Xinbote Chemical Co., Ltd., Tianjin, China) Transparent Mounting: Prior to xylene clearing and mounting, both the front and back sides of the sections were examined under a microscope (Olympus Sales & Service Co., Ltd., Tokyo, Japan). Subsequently, the sections were baked at 65 °C for one hour to enhance adhesion to the slides and to remove residual solvents. The dewaxing process was completed through two 20-min xylene treatments to ensure thorough removal of wax. Afterward, neutral resin (Solarbio Science & Technology Co., Ltd., Beijing, China) mounting was performed directly, followed by microscopic observation of the sections. This method aims to facilitate clear observation of varying pigmentation conditions in the tissues while minimizing interference from various dyes.

Quantitative analysis of hair shaft cross-sectional images from xylene sections was also conducted using ImageJ software (v1.37c) [10]. Following the conversion of color images to 8-bit grayscale, the pigmented regions (indicated by low grayscale values) were meticulously outlined manually, and their areas (Area) were recorded. The degree of pigmentation was quantified as the percentage of the pigmented area relative to the total cross-sectional area of the hair shaft (Area all = 100). The grading criteria were established as follows: symmetrical ≤ 1%, asymmetrical > 1% (*p* < 0.0001). Ten independent hair follicles were analyzed per sample, with data presented as mean ± standard error of the mean (SEM). Intergroup comparisons were performed using unpaired *t*-tests.

HE-stained sections were examined using an optical microscope. Quantitative analysis of the longitudinal sections of hair follicles was conducted with ImageJ software. To evaluate the symmetry of pigment deposition, the center of the dermal papilla served as the central boundary to delineate the ‘inner’ and ‘outer’ sides of the hair follicle [8]. Manual segmentation tools were employed to accurately select the inner and outer regions of pigment deposition within the hair bulb, where pigmentation was most pronounced. The color images were converted to 8-bit grayscale, with pigment deposition manifesting as low-gray-value regions. The percentage difference in area between the two regions was calculated using the formula: Percentage area difference = |Area_in − Area_out|/[(Area_in + Area_out)/2] × 100%. The criteria are: symmetric: mean area difference percentage < 50% (*p* < 0.05); asymmetric: mean area difference percentage ≥ 50% (*p* > 0.05). Ten independent hair follicles were analyzed for each sample. Data were presented as mean ± standard error of the mean (SEM), and intergroup comparisons were conducted using unpaired *t*-tests.

Primer validation: The qRT-PCR primers we designed for *TBX3* (*TBX3*-F: TCCTCCACGCTCTCCTCCAG, *TBX3*-R: TCCAAGCCGCTGACCAACC) and the reference gene *GAPDH* (*GAPDH*-F: GGCGATGCTGGTGCTGAATATG, *GAPDH*-R: AGCAGAAGGAGCAGAGATGATGAC) all adhere to the principle of spanning exon-exon junctions to effectively avoid genomic DNA amplification. Prior to formal experiments, we validated the amplification efficiency and specificity of the primers through preliminary tests. The amplification efficiency was calculated through standard curves to ensure it remained within the ideal range of 90–110%. Product specificity was confirmed by melt curve analysis, which required a single sharp peak, indicating specific amplification products and effectively excluding interference from non-specific amplifications such as primer dimers. Stability assessment of the reference gene (*GAPDH*): We recognize that the expression stability of reference genes may vary across different tissues, developmental stages, or experimental treatments. In this study, *GAPDH* was selected as the reference gene. Although *GAPDH* exhibits stable expression in many tissues, to ensure quantification accuracy, we validated its stability in the experimental samples by comparing Ct values across different experimental groups (such as different coat color regions) and calculating their standard deviation (SD). The Ct value fluctuations were within an acceptable range, meeting the requirements for use as a reference gene.

The images of immunohistochemically stained sections were subjected to semi-quantitative analysis using ImageJ software. In multiple randomly selected fields from each sample, we measured the integrated density and mean gray value of positively stained areas. To evaluate staining intensity, we established a semi-quantitative scoring system: a mean gray value greater than 150 was classified as weakly positive (1+), a mean gray value between 100 and 150 as moderately positive (2+), and a mean gray value of 100 or less as strongly positive (3+). These threshold values were determined based on preliminary experimental analyses of the samples in this study, ensuring that the scoring system effectively distinguished the observed variations in staining intensity within our experiments.

All experimental results in this study are based on at least three independent repetitions, and statistical analysis was conducted accordingly. We conducted a quantitative analysis of the grayscale values in stained sections utilizing WCIF ImageJ 1.37c software. Subsequently, all data were statistically analyzed using GraphPad Prism 10 software. For multiple group comparisons, we initially performed one-way ANOVA. When the one-way ANOVA results indicated significant differences between groups (*p* < 0.05), we proceeded with post hoc tests to identify which specific groups exhibited differences. Given that all groups in this study had equal sample sizes (*n* = 3), we selected Tukey’s honestly significant difference (HSD) test for multiple comparisons, which is particularly suitable for such cases. This method is widely employed to conduct pairwise comparisons of all possible group mean pairs following a significant ANOVA result, while strictly controlling the family-wise error rate. For pre-planned specific pairwise comparisons, we utilized unpaired *t*-tests with Bonferroni correction to adjust the significance level (α), accounting for the increased risk of false positives due to multiple comparisons. Specifically, the original significance level (α = 0.05) was divided by the number of comparisons performed to obtain the corrected significance threshold. In this study, the significance levels for all statistical tests were established as follows: a corrected *p* < 0.05 was deemed statistically significant (*), *p* < 0.01 as highly significant (**), *p* > 0.05 as non-significant (ns). All figures in the text are labeled with corrected p* values.

## 3. Results

### 3.1. Xylene Transparent Mounting Results

**Figure 3:**
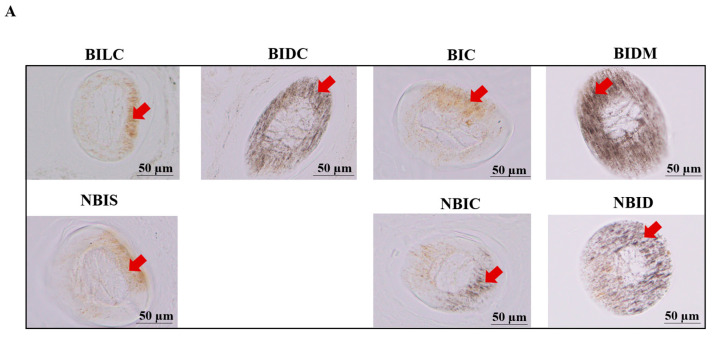

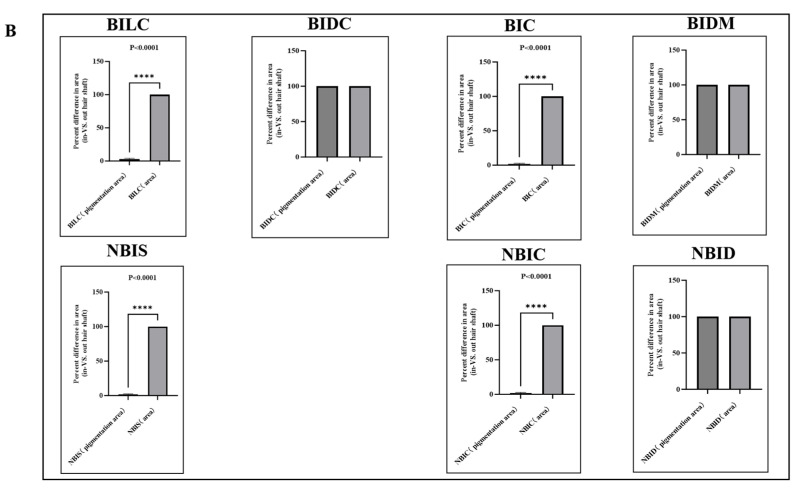
Xylene-cleared images of pigment deposition in cross-sections of hair shafts from different body parts of Bider horses and non-Bider horses. Note: Pigment deposition image (**A**) and quantitative results (**B**). The red arrows indicate the locations of pigment deposition (eumelanin). Bider horses *n* = 3, non-Bider horses *n* = 3. The scale used in the illustrations is 50 µm. The significance level is set as follows: *p* < 0.05 (****) indicates significant difference.

The examination of hair shaft cross-sections using the xylene clearing method (Figure 3) revealed distinct regional specificity in the distribution of pigment granules. In the dorsal midline (BIDM) and dark shoulder (BIDC) regions of Bider horses, as well as the dorsal midline (NBID) regions of non-Bider horses, pigment granules exhibited the densest and most uniformly symmetrical distribution patterns. In stark contrast, the light shoulder (BILC) and hip (BIC) regions of Bider horses, along with the shoulder (NBIS) and hip (NBIC) regions of non-Bider horses, demonstrated marked asymmetry, with pigment granules predominantly aggregating on one side of the hair shaft. Quantitative analysis further corroborated these morphological observations. The data indicate that the distribution symmetry in the BIDM, BIDC, and NBID regions is equal to 1%, reflecting highly symmetrical pigment deposition in these areas. In contrast, significant asymmetry (asymmetry > 1%) was detected in the BILC, BIC, NBIC, and NBIS regions, with extremely high statistical significance (*p* < 0.0001). For detailed data, please refer to the attachment (Supplementary Figure S3).

### 3.2. HE Staining Results

**Figure 4:**
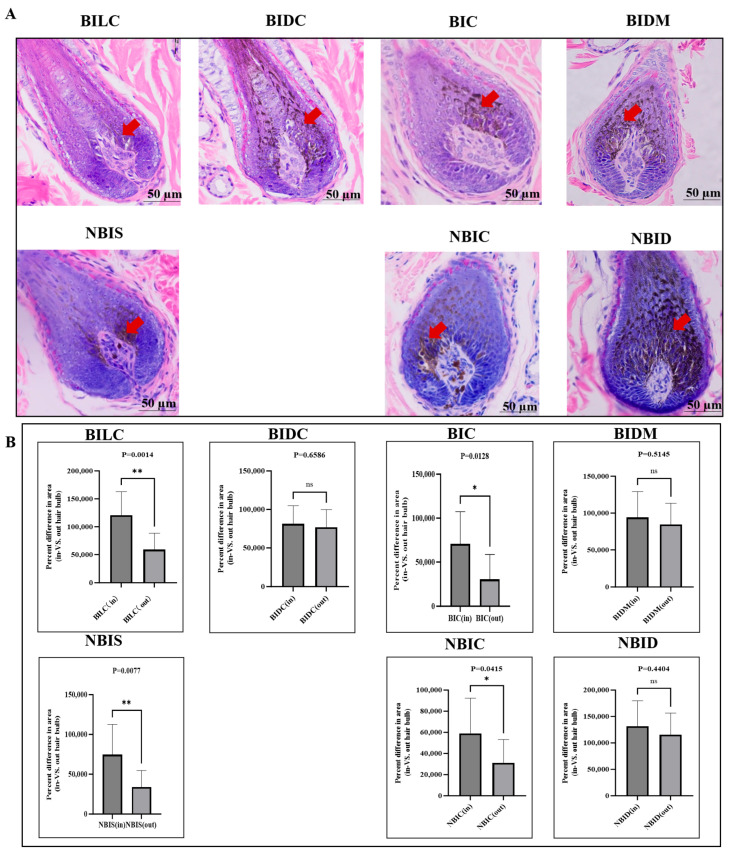
HE staining images of pigment deposition in the growing hair bulbs of longitudinal sections from different parts of Bider and non-Bider horses. Note: Pigment deposition image (**A**) and quantitative results (**B**). The red arrows indicate the locations of pigment deposition (eumelanin). Bider horses *n* = 3, non-Bider horses *n* = 3. The scale used in the illustrations is 50 µm. The significance level is set as follows: *p* < 0.05 (*/**) indicates significant difference, *p* > 0.05 (ns) indicates non- significant difference.

The HE staining results further confirmed the presence of significant regional specificity in pigment deposition (Figure 4). Morphological observations revealed that in the dorsal midline (BIDM) and dark shoulder (BIDC) regions of Bider horses, as well as the dorsal midline (NBID) region of non-Bider horses, the pigment cells in longitudinal sections of growing hair bulbs exhibited a typical symmetrical distribution pattern centered around the dermal papilla. To investigate the relationship between the symmetry of pigment deposition, we conducted a Pearson correlation analysis (Supplementary Figure S4). Quantitative analysis provided statistical support for these observations, with percentage differences in mean area between groups for these symmetrically distributed regions all being less than 50%, indicating no statistical significance (BIDM, *p* = 0.5145; BIDC, *p* = 0.6586; NBID, *p* = 0.4404). In stark contrast, areas exhibiting the tan trait, such as the light-colored shoulder (BILC) and hip (BIC) regions of Bider horses, as well as the shoulder (NBIS) and hip (NBIC) regions of non-Bider horses, displayed markedly uneven pigment cell deposition with asymmetric distribution. Quantitative analysis revealed that the mean percentage area difference in these regions equaled or exceeded 50%, demonstrating statistically significant differences (BILC, *p* = 0.0014; BIC, *p* = 0.0128; NBIC, *p* = 0.0415; NBIS, *p* = 0.0077). For detailed data, please refer to the attachment (Supplementary Figure S4).

### 3.3. RT-qPCR Results

**Figure 5:**
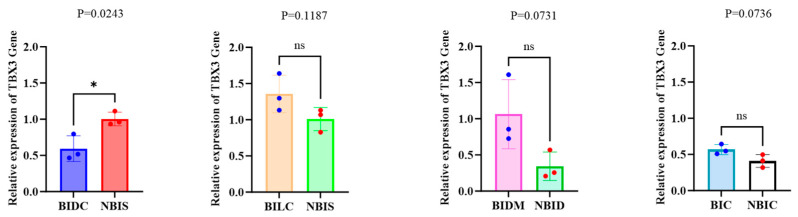
Relative expression levels of the *TBX3* gene in skin tissues from different sampling sites of Bider and non-Bider horses. Note: Error bars indicate the standard error of the mean (SEM). Bider horses *n* = 3, non-Bider horses *n* = 3. The significance level is set as follows: *p* < 0.05 (*) indicates significant difference; *p* > 0.05 (ns) indicates no significant difference. The four comparison groups in the figure are represented by blue and red circles for data points from different parts.

The purity and concentration of RNA were 175 assessed using an enzyme marker, with acceptable purity indicated by a 260/280 ratio of 176 1.9 to 2.2 and integrity confirmed by RIN values between 6.3 and 7.4. Analysis of *TBX3* mRNA expression levels revealed significant differences across various regions of equine skin (Figure 5). To investigate the relationship between *TBX3* expression levels we conducted a Pearson correlation analysis (Supplementary Figure S5). Specifically, the *TBX3* mRNA expression in the shoulder (NBIS) tissue of non-Bider horses was significantly higher than that in the dark-colored shoulder (BIDC) tissue of Bider horses (*p* = 0.0243). In the remaining three comparisons (shoulder (NBIS) tissue of non-Bider horses vs. light-colored shoulder (BILC) tissue of Bider horses, dorsal midline (NBID) of non-Bider horses vs. dorsal midline (BIDM) of Bider horses, and croup (NBIC) of non-Bider horses vs. croup (BIC) of Bider horses), although the differences did not reach statistical significance (*p* > 0.05), the expression levels in the dorsal midline (BIDM) and croup (BIC) tissues of Bider horses still showed a trend of being higher than those in the corresponding tissues of non-Bider horses.

### 3.4. Western Blot Results

**Figure 6:**
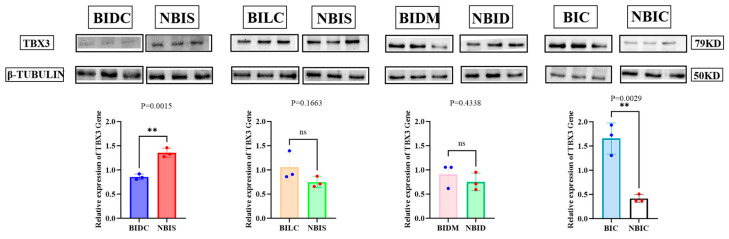
Relative expression levels of *TBX3* protein in skin tissues from different sampling sites of Bider and non-Bider horses. Note: The bar graph illustrates the relative expression levels of *TBX3* protein in skin tissues from different regions, with different colors representing different body parts. Error bars indicate the standard error of the mean (SEM). Bider horses *n* = 3, non-Bider horses *n* = 3. The significance levels are set as follows: *p* < 0.01 (**) indicates highly significant differences, and *p* > 0.05 (ns) indicates no significant differences. The four comparison groups in the figure are represented by blue and red circles for data points from different parts.

Western Blot analysis revealed differential markings of *TBX3* protein expression across various regions (Figure 6). To investigate the relationship between *TBX3* expression levels we conducted a Pearson correlation analysis (Supplementary Figure S6). The expression of *TBX3* protein in the non-Bider horse shoulder (NBIS) was significantly higher than that in the dark-colored (BIDC) Bider horse shoulder (*p* = 0.0015), while the expression of *TBX3* protein in the Bider horse croup (BIC) was significantly higher than that in the non-Bider horse croup (NBIC) (*p* = 0.0029). The remaining differences were not statistically significant (*p* > 0.05).

### 3.5. Protein Localization Results

**Figure 7:**
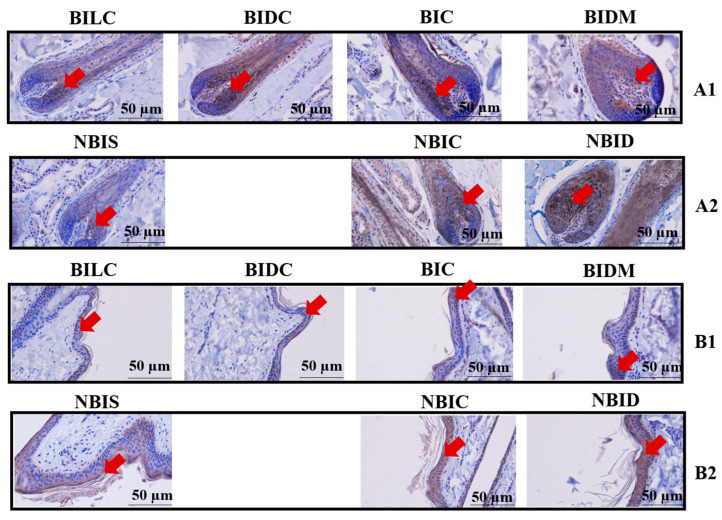
Immunohistochemical staining of *TBX3* protein in skin tissues from different sampling sites of Bider and non-Bider horses. Note: Y Localization of *TBX3* protein in the hair bulb region (**A1**,**A2**); Localization of *TBX3* protein in the epidermis (**B1**,**B2**). Bider horses *n* = 3, non-Bider horses *n* = 3. Red arrow shows the location of pigment deposition. The scale used in the illustrations is 50 µm.

Immunohistochemical staining results indicated that *TBX3* protein was localized in the hair bulb and epidermal layers of both Bider and non-Bider horse breeds. In the anagen hair follicles, the levels of *TBX3* protein deposition in the dorsal midline (NBID) and shoulder region (NBIS) of non-Bider horses were significantly higher than those in the experimental Bider horse group (*p* < 0.01). However, no statistically significant differences were observed in the croup group (Figure 7(A1,A2)/Supplementary Figure S7). In epidermal tissues, *TBX3* protein expression exhibited statistically significant differences exclusively in the croup group, with higher levels in non-Bider horses compared to Bider horses (*p* < 0.05), while no significant differences were detected in the other three groups (Figure 7(B1,B2)/Supplementary Figure S7).

## 4. Discussion

The diversity of coat colors arises from the synergistic effects of different genes, which create a complex regulatory network [13,14]. The production and absence of melanin form the biochemical foundation of mammalian coat coloration. This process is regulated not only by intricate gene interactions but is also significantly influenced by environmental factors such as light intensity, seasonal variations, and nutritional status [15]. Most coat color characteristics in domestic horses result from long-term human-directed selective breeding. In contrast, wild-type coat colors more prominently reflect the impact of natural selection and evolution. Through distinctive coat markings—such as dorsal stripes, leg barring, and shoulder striping—these animals adeptly blend into their natural environments, fulfilling various ecological functions including predator avoidance, efficient foraging, mate attraction, and UV radiation resistance, thereby demonstrating high environmental adaptability [16]. Among these coat colors, the Dun coloration is widely regarded as a wild-type characteristic, commonly observed in wild equids such as the Asiatic wild ass and Przewalski’s horse. This coloration typically features clearly visible dorsal stripes, prominent leg barring, and shoulder striping [9]. Mongolian horses, having not undergone intensive artificial selection, continue to thrive in semi-wild conditions, thereby retaining the genetic diversity of their ancestors [17]. Reports indicate that the Bider marking has been identified in Przewalski’s horses and Mongolian horses [12]. This trait typically manifests in diluted coat colors such as blue dun, yellow dun, and red dun. Research indicates that the *TBX3* gene plays a significant role in the formation of dun coat color in domestic horses [8], with mutations in the *TBX3* gene leading to the development of dorsal stripes and asymmetric pigment deposition on the rump in dun coats.

Our preliminary study focused on the Dun Mongolian horses with Bider markings as the research subjects. Through multidisciplinary technical approaches, it initially explored the differential expression of the *TBX3* gene across various skin regions (shoulder, dorsal midline, and croup) of the same individual. The findings revealed that the Bider-marked area (dark-colored shoulder) and the dorsal midline skin exhibited a symmetrical distribution of pigment deposition in hair bulbs, while the croup and the Bider-marked area (light-colored shoulder) showed significant asymmetry. The mRNA expression level of the *TBX3* gene was significantly higher in the croup compared to the shoulder and dorsal midline, which was consistent with the Western blot results. Interestingly, *TBX3* mRNA and protein expression in the dark-colored shoulder was found to be higher than in the light-colored shoulder. Immunohistochemical analysis demonstrated that *TBX3* protein was primarily localized in the hair bulb and epidermal regions [9].

To further investigate the expression and localization differences of *TBX3* between Dun Mongolian horses with and without the Bider marking, we conducted an extended study. The staining results revealed that in the Bider horses, the pigment deposition around hair follicles in the light-colored areas of the croups and shoulders exhibited significant asymmetry, while the pigment deposition in the dorsal midline and dark-colored shoulder areas appeared relatively uniform and symmetrical. This finding aligns with the research results of Imsland [8] and Tana [9], further confirming the complexity and diversity of pigment deposition in equine skin tissues. In non- Bider horses, the pigment deposition markings were relatively consistent across different body regions, with no significant differences observed. Asymmetrical pigment deposition is not uncommon in the animal kingdom; however, its specific mechanisms in equine skin tissues have not yet been fully elucidated. The results of this study suggest that this asymmetry may be related to the morphology and distribution of hair follicles, as well as the associated gene expression markings. As an important component of skin tissue, hair follicles not only participate in hair growth and cycle regulation but may also influence the distribution and function of pigment cells [18]. Therefore, further exploration of the correlation between hair follicle morphology and pigment deposition is of great significance for understanding the biological characteristics of equine skin tissue. Additionally, in non-Bider horses, pigment deposition is relatively consistent across different body regions, with no significant variations. This finding suggests that the skin tissue of non-Bider horses may possess a more uniform regulatory mechanism for pigment deposition. However, this does not imply that the skin tissue of non-Bider horses lacks diversity in gene expression and functionality. On the contrary, as research progresses, more genes and pathways specifically associated with non-splashed white horse skin tissue may be discovered.

Western blot analysis revealed significant differences in *TBX3* protein expression levels between Bider horses and non-Bider horses across various tissue regions. Notably, *TBX3* expression was consistently higher in all examined regions of Bider horses compared to their non-Bider counterparts, with the most pronounced difference observed in shoulder tissues. This finding uncovers a distinctive expression marking of *TBX3* in the skin tissues of Mongolian Bider horses, providing crucial insights for investigating its role in pigment deposition and hair follicle development. The *TBX3* gene, a well-documented transcription factor, has been shown to participate in developmental processes across multiple organisms [19]. Our study highlights this gene’s unique expression marking in Mongolian Bider horse skin tissues, suggesting its potential involvement in phenotype-specific processes such as pigment deposition and hair follicle development. Notably, we found the most significant expression of the *TBX3* gene in the rump region of Mongolian Bider horses, indicating its potential key role in hair follicle development at this site. An intriguing finding is that, although *TBX3* protein expression exhibits significant regional variations, no distinct differences were observed in its cellular localization between the hair bulb and epidermal layers, with only noticeable variations in staining intensity. These areas are critical for melanocyte activity and play vital roles in hair and skin pigmentation [20,21]. This suggests that *TBX3*’s function may primarily depend on its expression levels rather than its localization markings.

This study elucidates the intricate expression marking of *TBX3* in equine skin tissues. The most notable finding is that in dun horses, both *TBX3* mRNA and protein expression levels are generally elevated in light-colored areas compared to dark-colored regions. This observation contradicts conventional understanding; traditionally, it is believed that the ‘dun’ phenotype arises from loss-of-function alleles of *TBX3*, which would typically correlate with lower *TBX3* expression and consequently reduced pigment dilution (i.e., darker coloration). Moreover, even within light-colored areas, significant differences in *TBX3* expression are observed between the shoulder and croup regions, with higher levels in Bider horses compared to non-Bider horses, indicating the presence of more sophisticated region-specific regulatory mechanisms.

Methodological and technical factors are critical considerations that contribute to the seemingly contradictory results mentioned above. Studies indicate that while no statistically significant differences were observed among certain comparison groups, *TBX3* protein expression was notably higher in the croup region of horses with Bider markings. This observation may suggest variations in protein abundance across different anatomical sites or differences in antibody recognition efficiency at the protein level. For instance, post-translational modifications (e.g., phosphorylation) of the *TBX3* protein in specific regions might obscure or alter antibody-recognized epitopes, thereby affecting Western Blot detection signals. Additionally, the inherent heterogeneity in tissue sampling cannot be overlooked. Hair follicles in different growth cycle phases (anagen, catagen, telogen) exhibit distinct pigment synthesis activities and gene expression profiles. If hair follicles are not precisely matched to the same growth cycle during sampling, or if there are inherent differences in the cellular composition ratios (e.g., melanocytes to keratinocytes) of follicles from differently pigmented regions, substantial variability may be introduced. Particularly with small sample sizes, individual variations and sampling errors become magnified, complicating the detection of potentially subtle expression differences at statistically significant levels. This can even lead to results that deviate from the true biological scenario [9,10].

This study found that *TBX3* expression was, in fact, higher in lightly pigmented regions. This counterintuitive phenomenon challenges the simplistic linear model that posits “*TBX3* loss-of-function leads to pigment dilution.” Possible biological explanations for this observation include compensatory feedback mechanisms [22]. This study acknowledges certain limitations. Given that the Bider marking is extremely rare—observed in only 1% of Mongolian horse populations [12]—the sample size is relatively limited, and its genotype remains unknown. Future research could focus on several directions: expanding sample sizes to mitigate the impact of individual variations; employing in situ hybridization and immunohistochemistry to accurately localize mRNA and protein expression in tissues; and further analyzing *TBX3*’s specific isoforms and interacting proteins in different coat color regions. These studies will contribute to a more comprehensive elucidation of the intricate mechanisms by which *TBX3* regulates pigmentation.

This study confirms the association between the differential expression of the *TBX3* gene and phenotypic traits, while not excluding the possibility that inherent regional differences in skin structure (e.g., hair follicle density, melanocyte distribution) may contribute to the observed variations. More importantly, the key internal comparisons in horses exhibiting the ‘Bider’ marking (e.g., light-colored shoulders versus dark-colored dorsal midline) were conducted between adjacent regions of the same individual. This within-individual comparison helps control for most genetic and systemic environmental factors, thereby strengthening the argument that the differential expression of *TBX3* is associated with the pigment pattern itself, rather than merely with macro-anatomical differences between distant body regions. Additionally, the upstream regulatory mechanisms and downstream signaling pathways remain unclear, suggesting the probable existence of upstream regulatory factors. Future research should focus on exploring the interaction network between the *TBX3* gene and other coat color-related genes to elucidate the complete genetic map underlying the formation of Bider markings. Furthermore, it remains uncertain whether the *TBX3* gene directly participates in determining Bider markings. Potential differences in the cellular composition of skin samples (bulk analysis versus single-cell expression profiling) may also influence the research findings. Subsequent studies should conduct whole-transcriptome analysis rather than being confined solely to *TBX3* gene expression research.

## 5. Conclusions

*TBX3* exhibits low expression in the dark-colored shoulder and high expression in the croup. Its region-specific expression differences are significantly correlated with the symmetry of pigment deposition and may be involved in the formation of Bider markings.

## Data Availability

The original contributions presented in the study are included in the article; further inquiries can be directed to the corresponding authors.

## Supplementary Materials

The following supporting information can be downloaded at: https://www.mdpi.com/article/10.3390/ani16020297/s1, Figure S1: Different primitive markings formed on a dun Mongolian horse; Figure S2: Example of sample collection from a dun Bider horse and a dun non-Bider horse; Figure S3: Xylene-cleared images of pigment deposition in cross-sections of hair shafts from different body parts of Bider horses and non-Bider horses; Figure S4: HE staining images of pigment deposition in the growing hair bulbs of longitudinal sections from different parts of Bider and non-Bider horses; Figure S5: Relative expression levels of the *TBX3* gene in skin tissues from different sampling sites of Bider and non-Bider horses, Figure S6: Relative expression levels of *TBX3* protein in skin tissues from different sampling sites of Bider and non-Bider horses; Figure S7: Immunohistochemical staining of TBX3 protein in skin tissues from different sampling sites of Bider and non-Bider horses.

## Abbreviations

The following abbreviations are used in this manuscript:

BILC	Bider light-colored shoulder Mongolian horses
BIDC	Bider dark-colored shoulder Mongolian horses
BIC	Bider croup Mongolian horses
BIDM	Bider dorsal midline Mongolian horses
NBIS	Non-Bider shoulder Mongolian horses
NBIC	Non-Bider croup Mongolian horses
NBID	Non-Bider dorsal midline Mongolian horses
