# Supplementary figures and images for: Differential Expression and Analysis of TBX3 Gene in Skin Tissues of Dun Mongolian Horses with and Without Bider Markings
Source: Animals (Basel). 2026 Jan 18;16(2):297. doi: 10.3390/ani16020297 (PMC12837385; doi:10.3390/ani16020297)

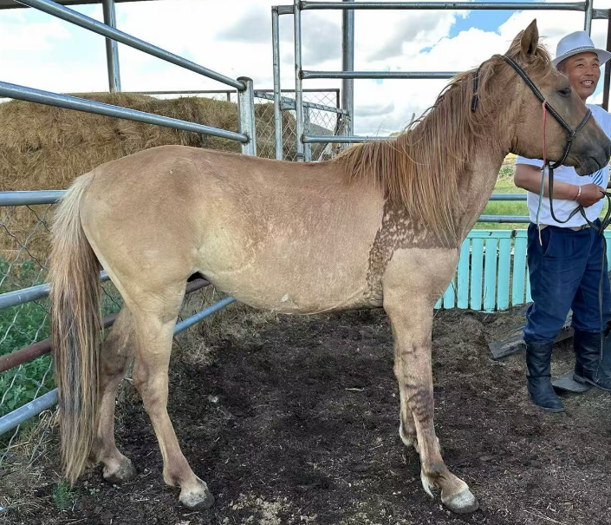

Supplement: Supplementary file 1 [file animals-16-00297-s001.zip › Figure S1/Bider horse.tif]

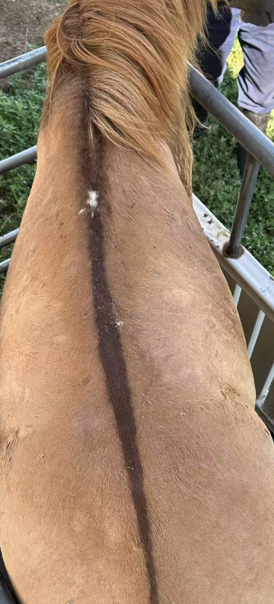

Supplement: Supplementary file 1 [file animals-16-00297-s001.zip › Figure S1/dorsal midline.tif]

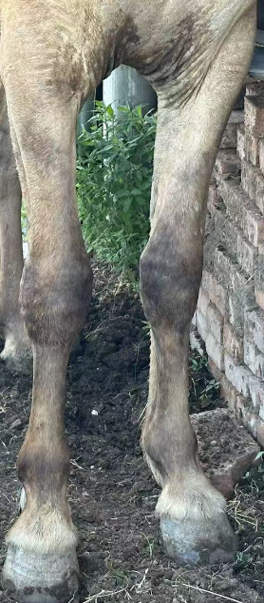

Supplement: Supplementary file 1 [file animals-16-00297-s001.zip › Figure S1/leg stripes.tif]

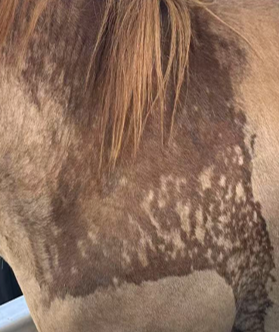

Supplement: Supplementary file 1 [file animals-16-00297-s001.zip › Figure S1/shoulder stripe(left).tif]

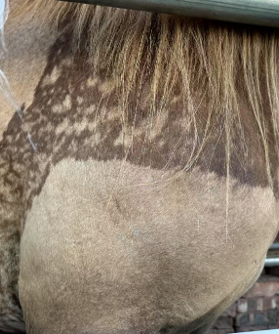

Supplement: Supplementary file 1 [file animals-16-00297-s001.zip › Figure S1/shoulder stripe(right).tif]

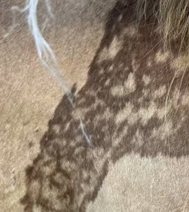

Supplement: Supplementary file 1 [file animals-16-00297-s001.zip › Figure S2/Bider shouder.tif]

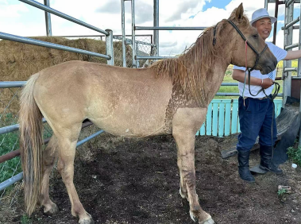

Supplement: Supplementary file 1 [file animals-16-00297-s001.zip › Figure S2/Bider.tif]

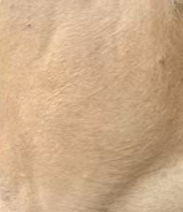

Supplement: Supplementary file 1 [file animals-16-00297-s001.zip › Figure S2/non Bider shouder.tif]

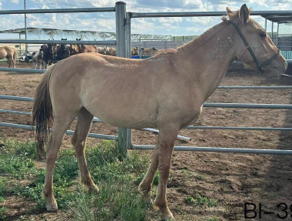

Supplement: Supplementary file 1 [file animals-16-00297-s001.zip › Figure S2/non Bider.tif]

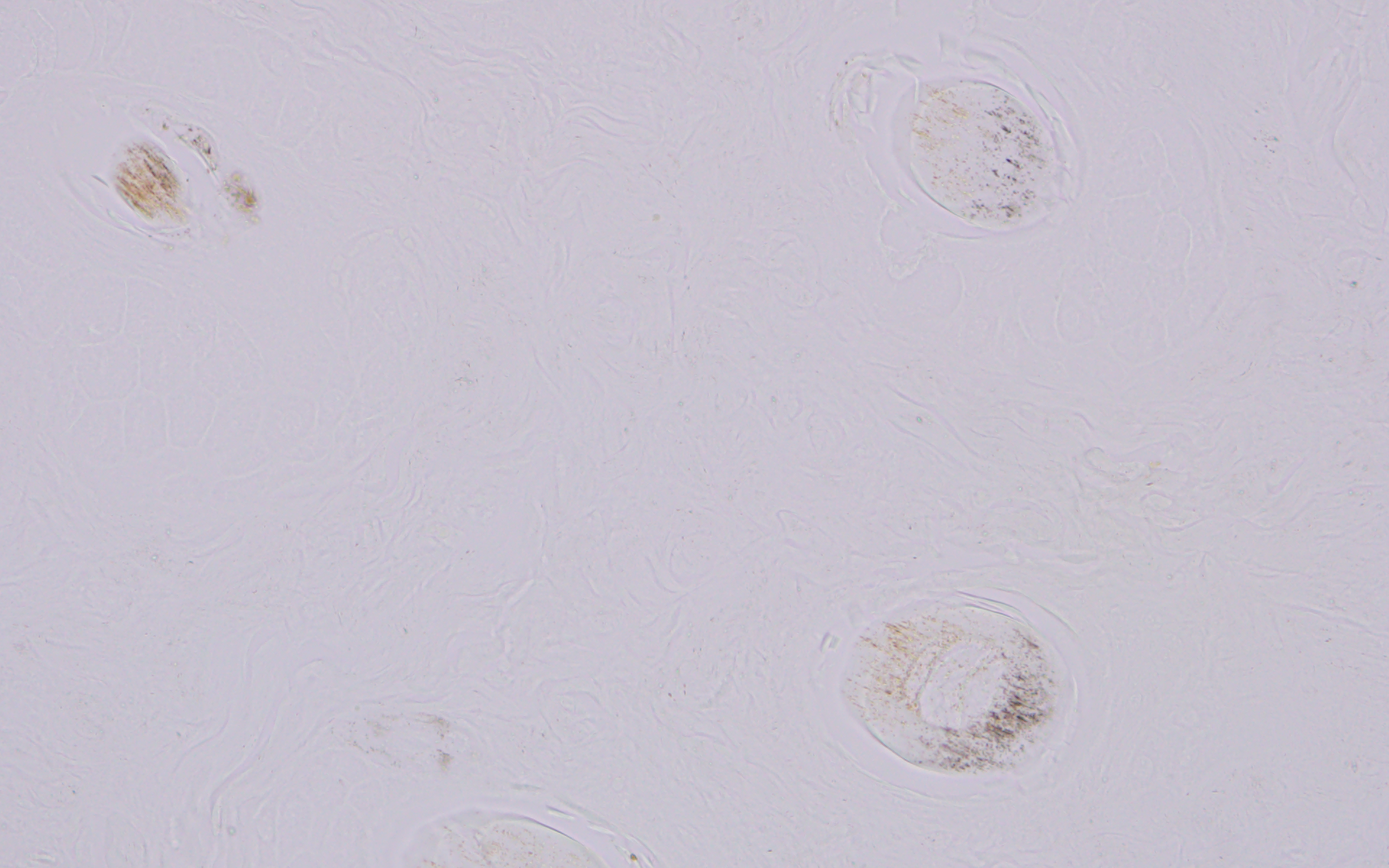

Supplement: Supplementary file 1 [file animals-16-00297-s001.zip › Figure S3/FYBC.tif]

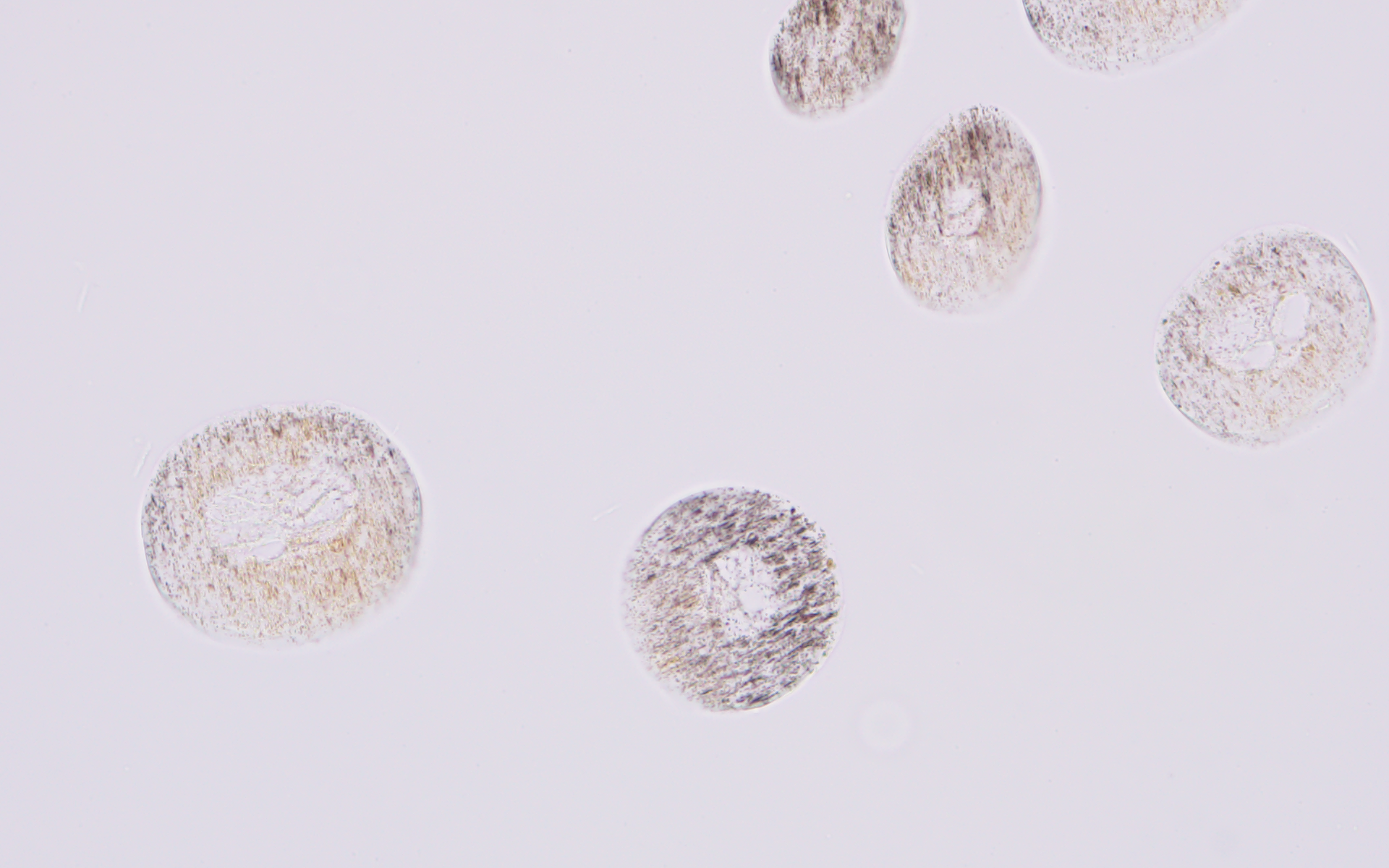

Supplement: Supplementary file 1 [file animals-16-00297-s001.zip › Figure S3/FYBD.tif]

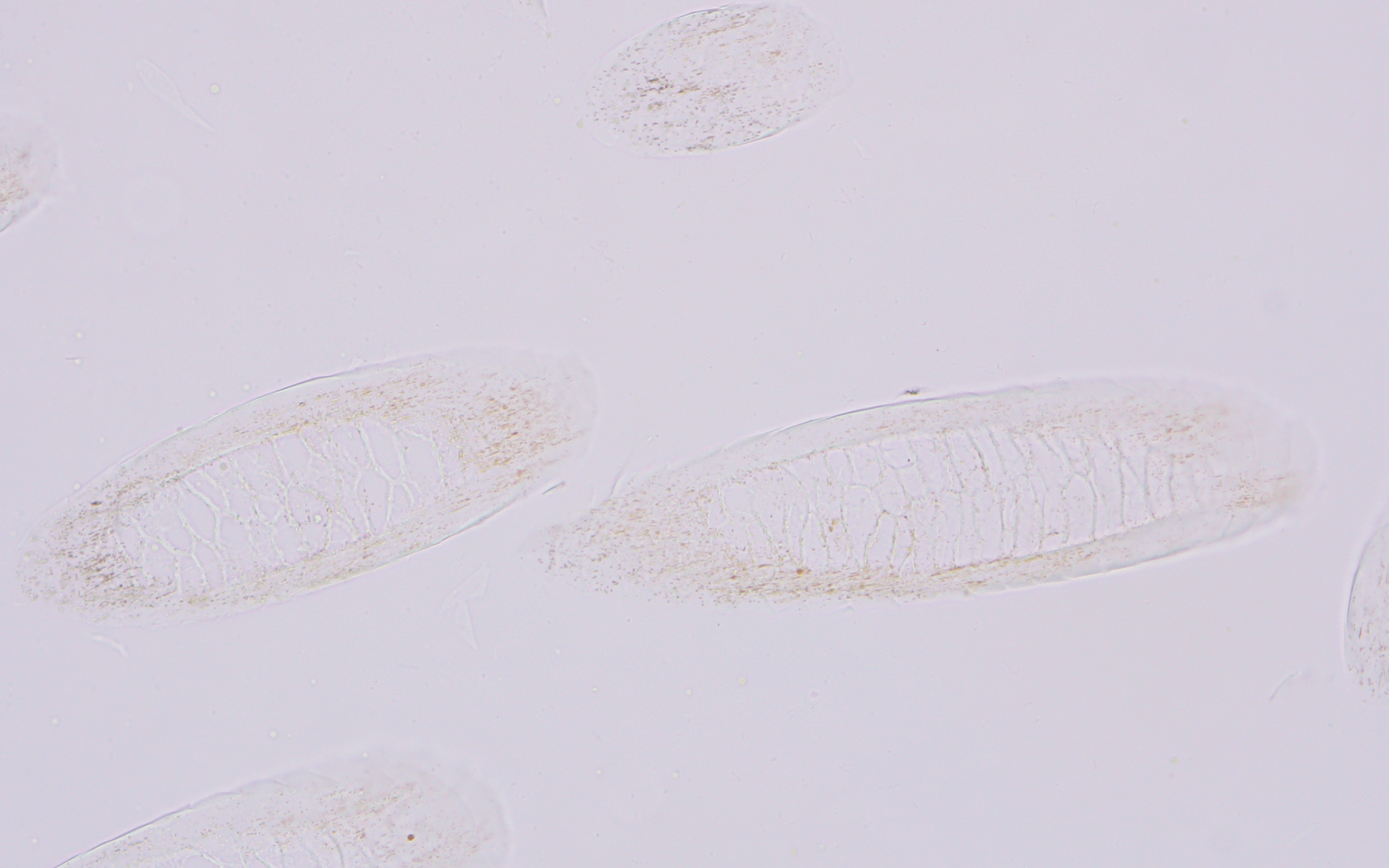

Supplement: Supplementary file 1 [file animals-16-00297-s001.zip › Figure S3/FYBS.tif]

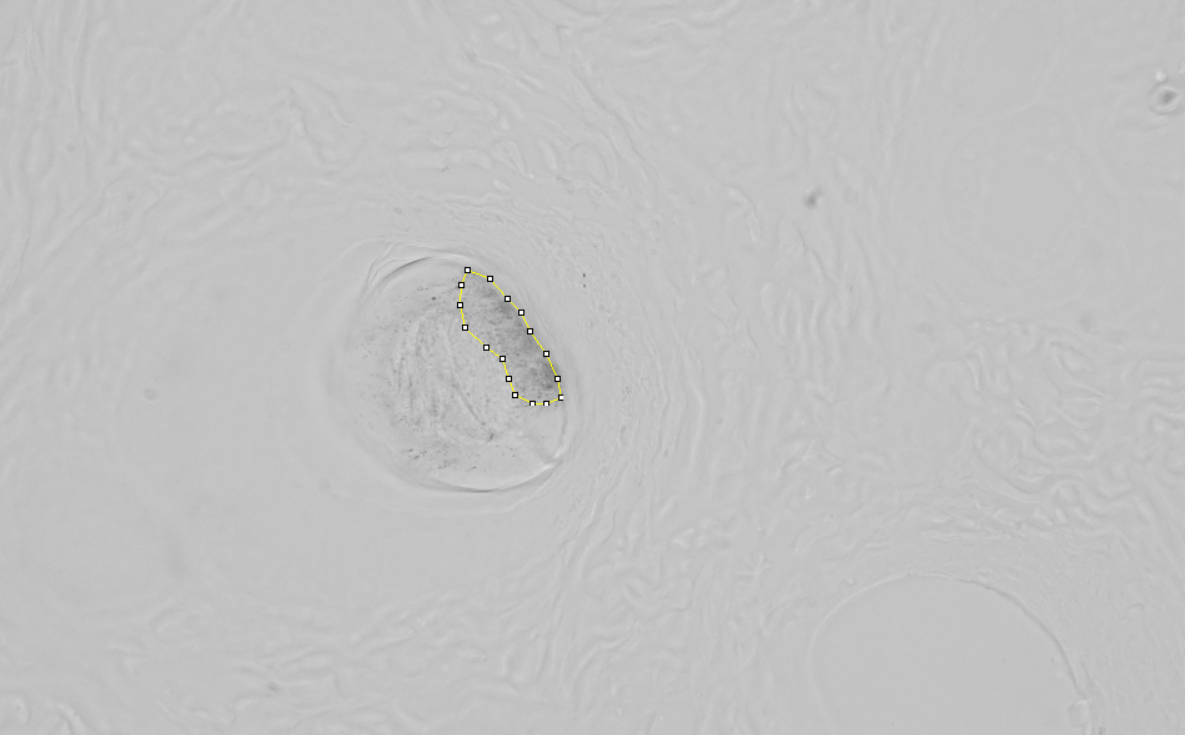

Supplement: Supplementary file 1 [file animals-16-00297-s001.zip › Figure S3/Xylene Transparent Mounting results/BIC/BIC xylene.png]

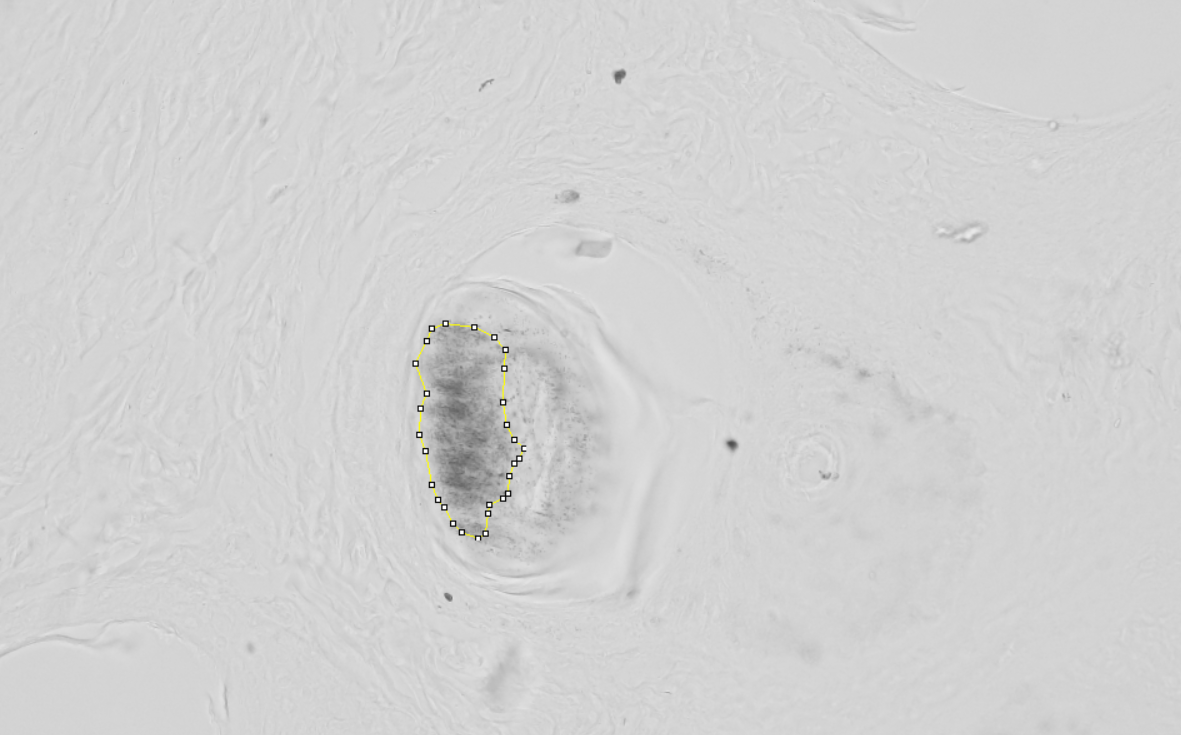

Supplement: Supplementary file 1 [file animals-16-00297-s001.zip › Figure S3/Xylene Transparent Mounting results/BILC/BILC xylene.png]

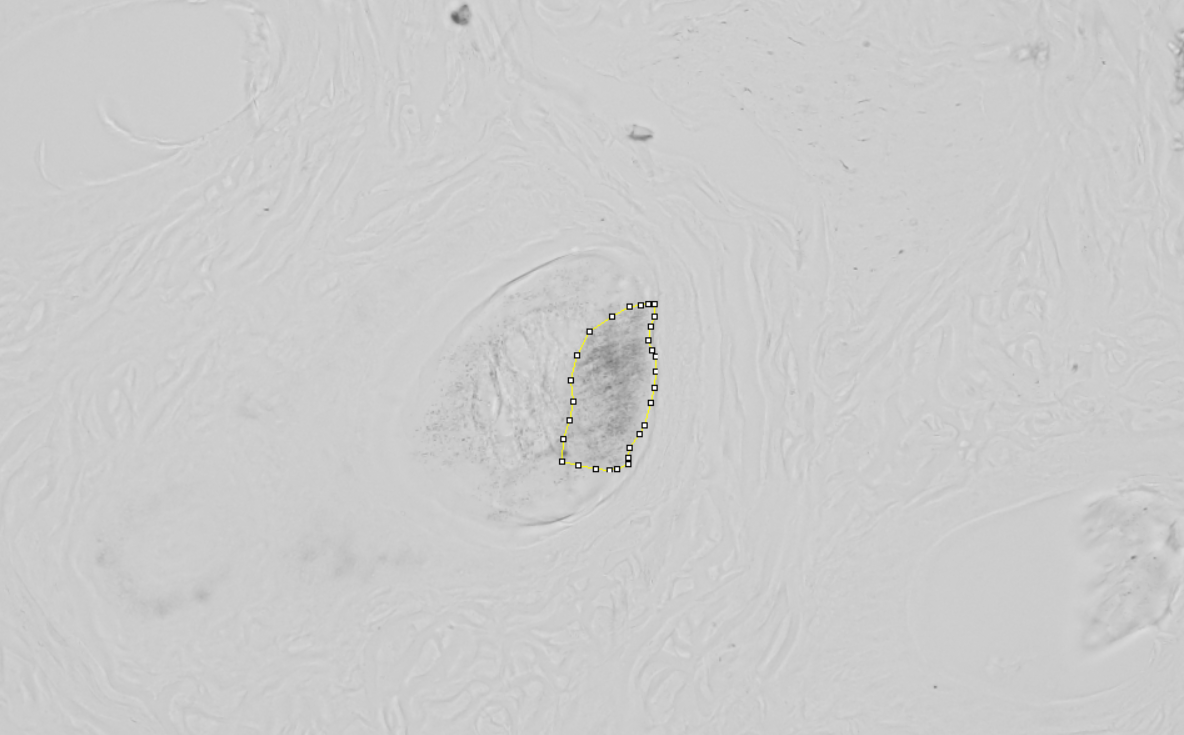

Supplement: Supplementary file 1 [file animals-16-00297-s001.zip › Figure S3/Xylene Transparent Mounting results/NBIC/NBIC xylene.png]

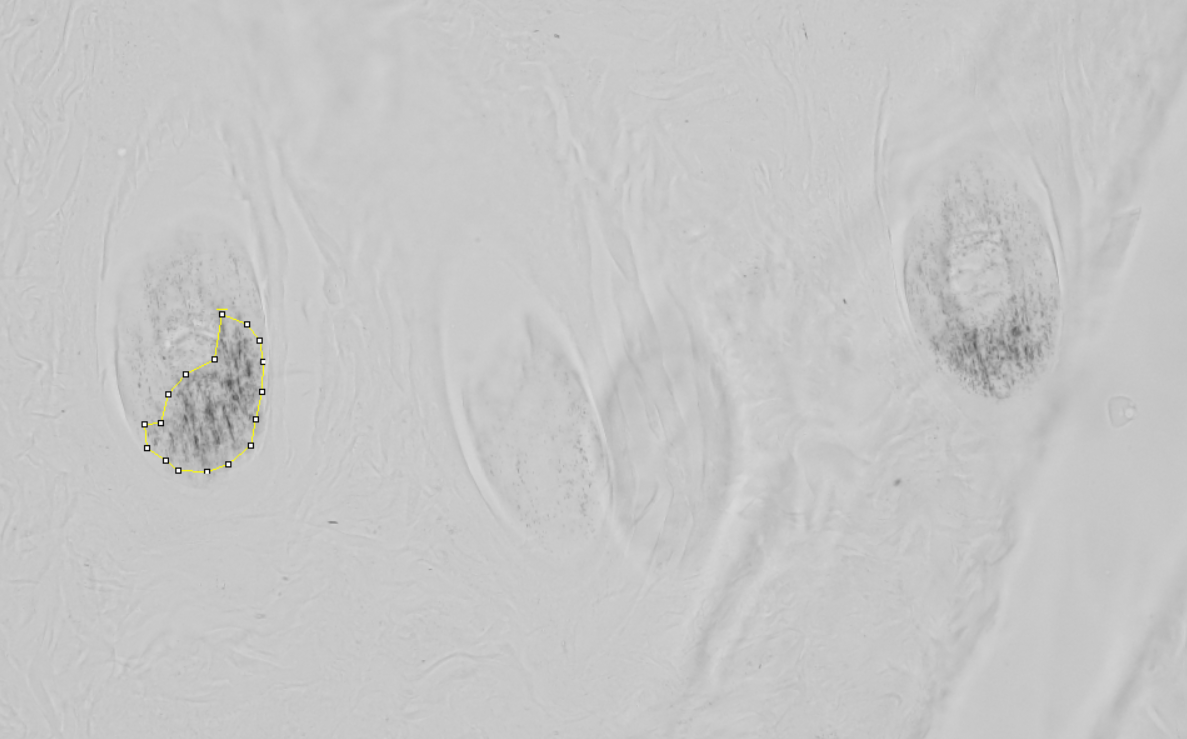

Supplement: Supplementary file 1 [file animals-16-00297-s001.zip › Figure S3/Xylene Transparent Mounting results/NBIS/NBIS xylene.png]

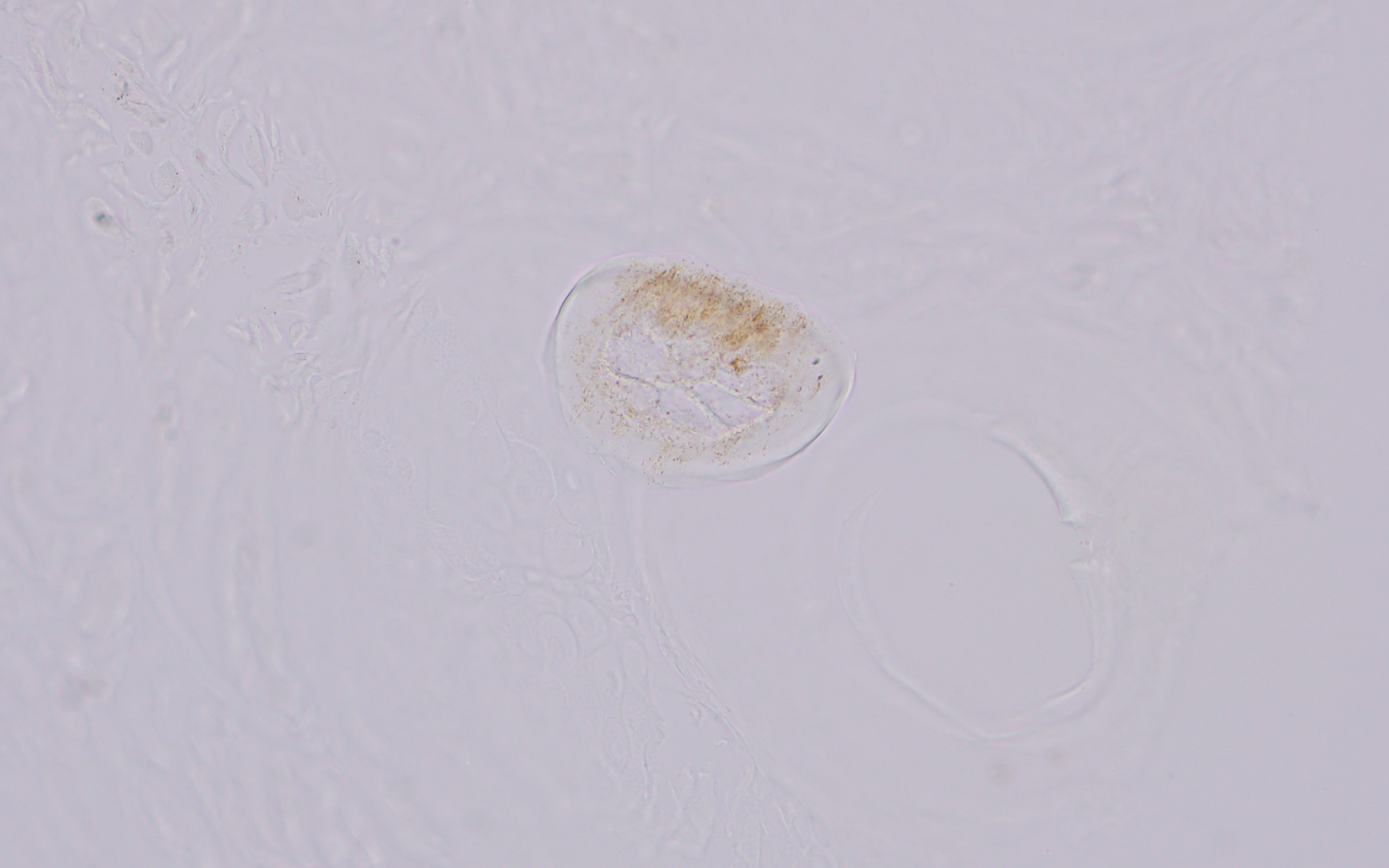

Supplement: Supplementary file 1 [file animals-16-00297-s001.zip › Figure S3/YBC.tif]

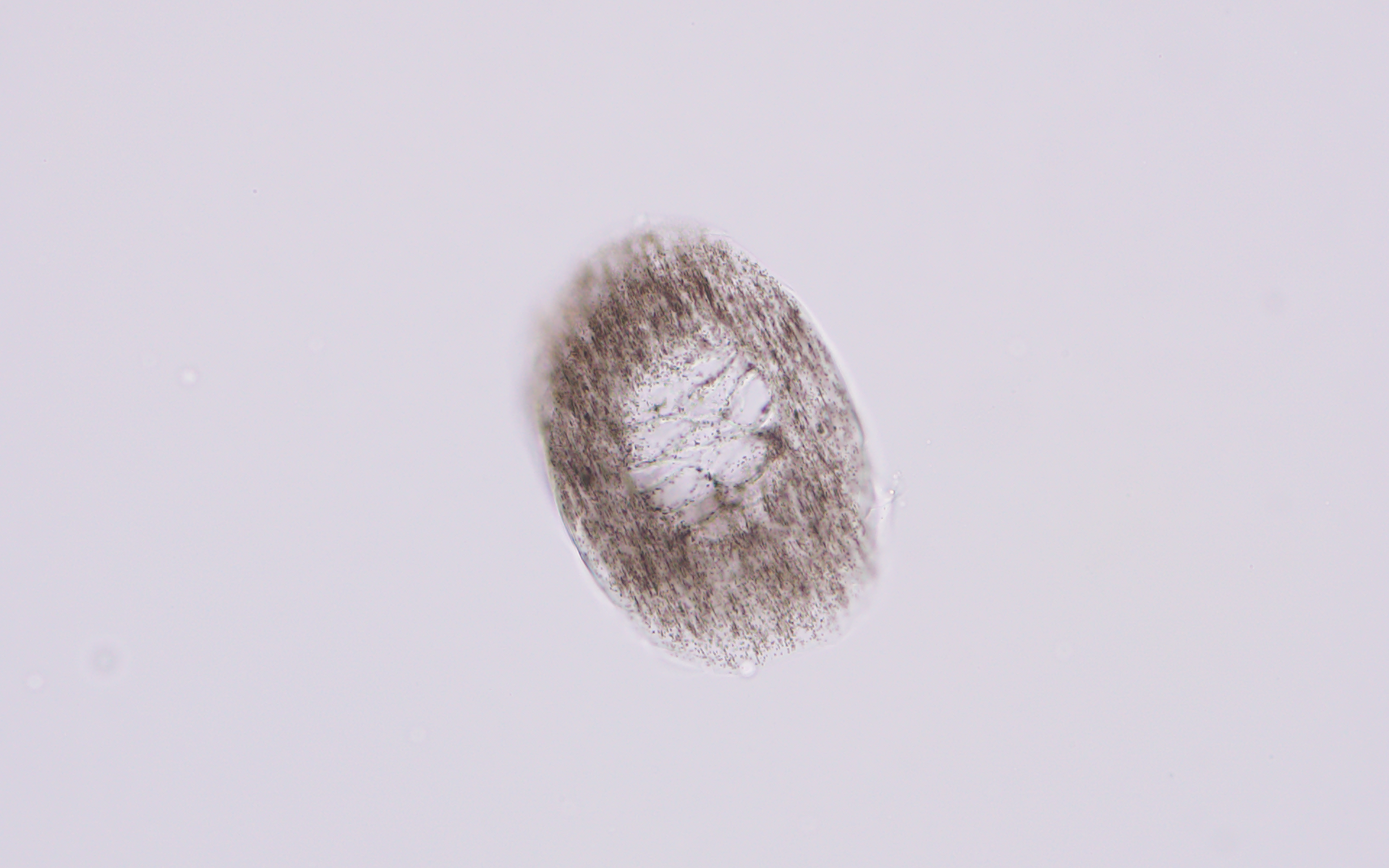

Supplement: Supplementary file 1 [file animals-16-00297-s001.zip › Figure S3/YBD.tif]

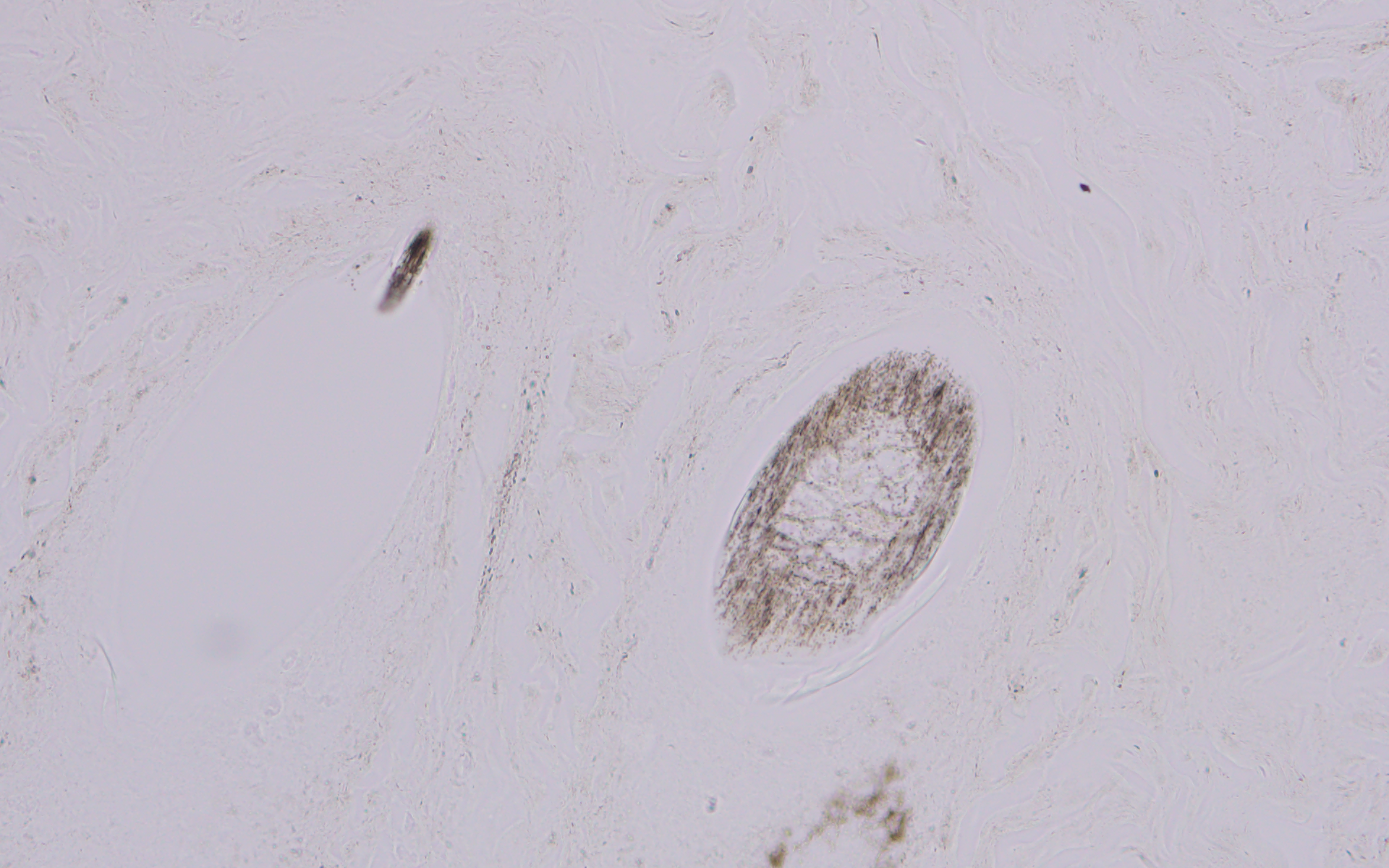

Supplement: Supplementary file 1 [file animals-16-00297-s001.zip › Figure S3/YBDC.tif]

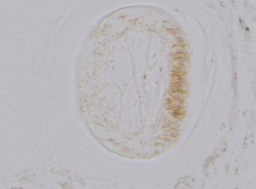

Supplement: Supplementary file 1 [file animals-16-00297-s001.zip › Figure S3/YBLC.tif]

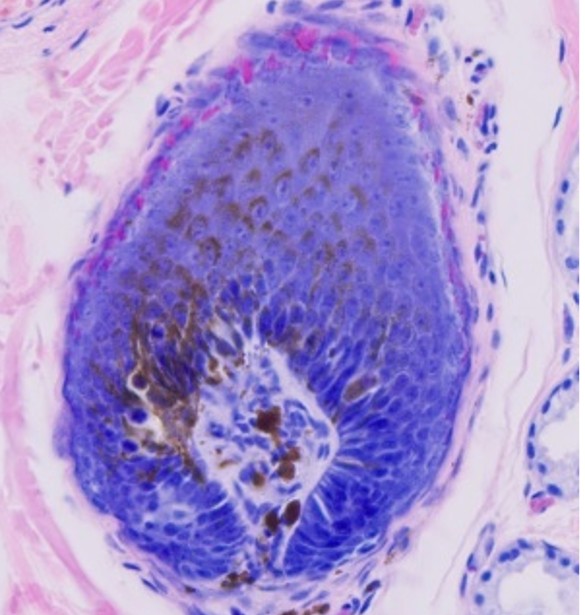

Supplement: Supplementary file 1 [file animals-16-00297-s001.zip › Figure S4/FYBC.jpg]

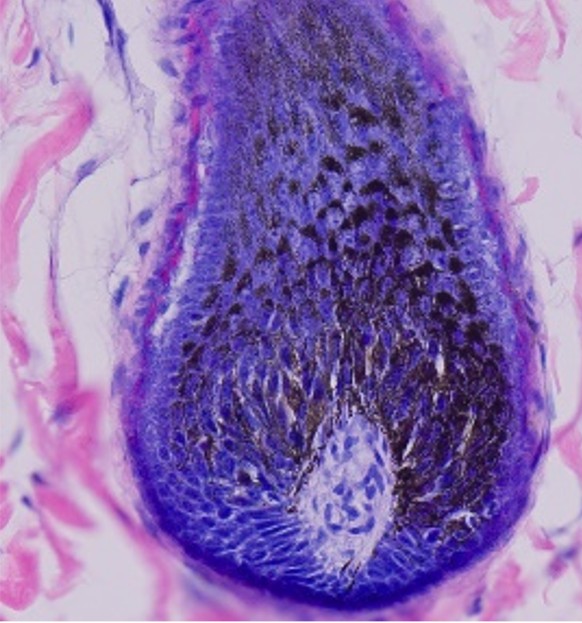

Supplement: Supplementary file 1 [file animals-16-00297-s001.zip › Figure S4/FYBD.jpg]

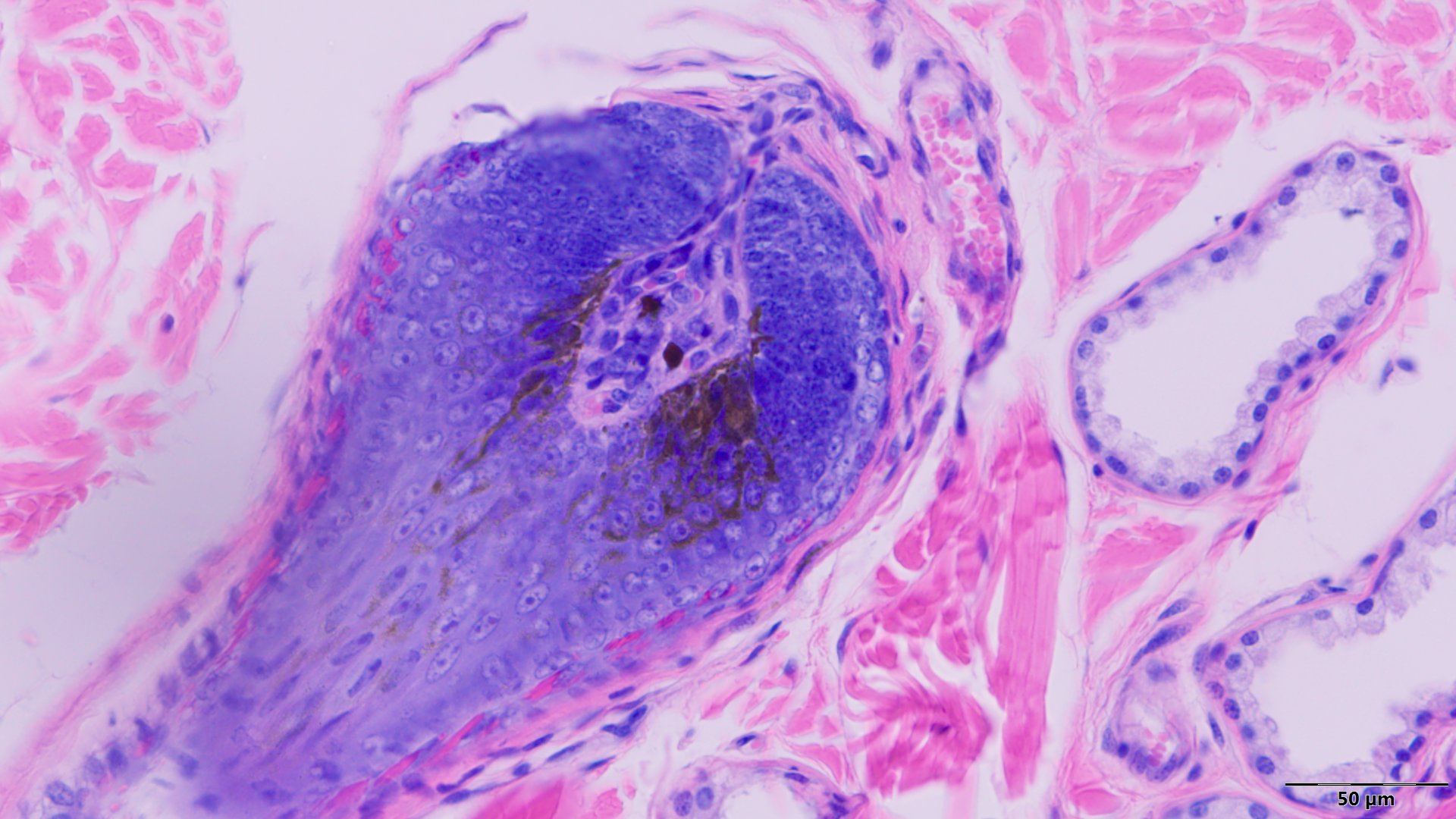

Supplement: Supplementary file 1 [file animals-16-00297-s001.zip › Figure S4/FYBS.jpg]

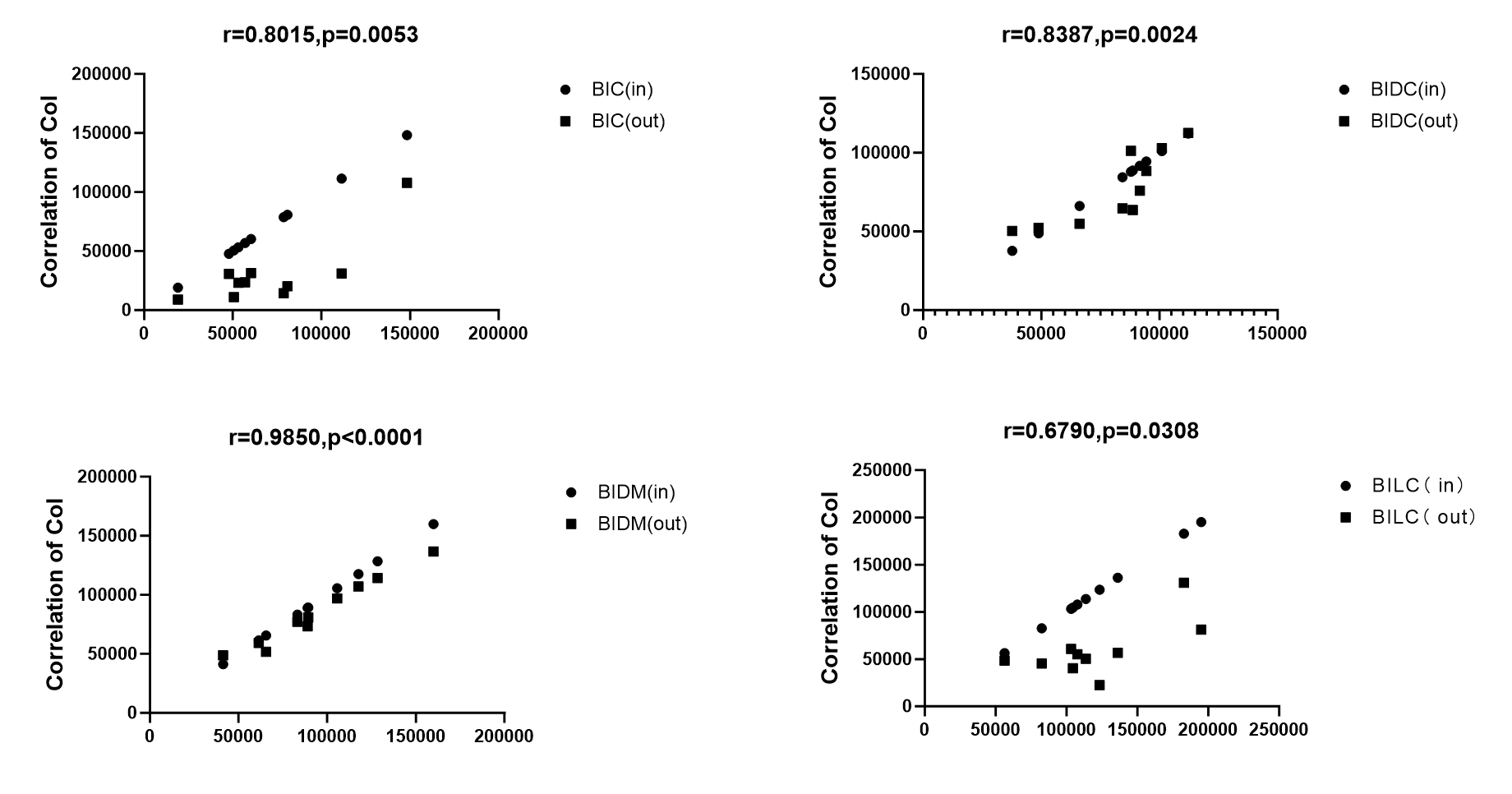

Supplement: Supplementary file 1 [file animals-16-00297-s001.zip › Figure S4/HE Staining Results/BI pigment deposition symmetry.png]

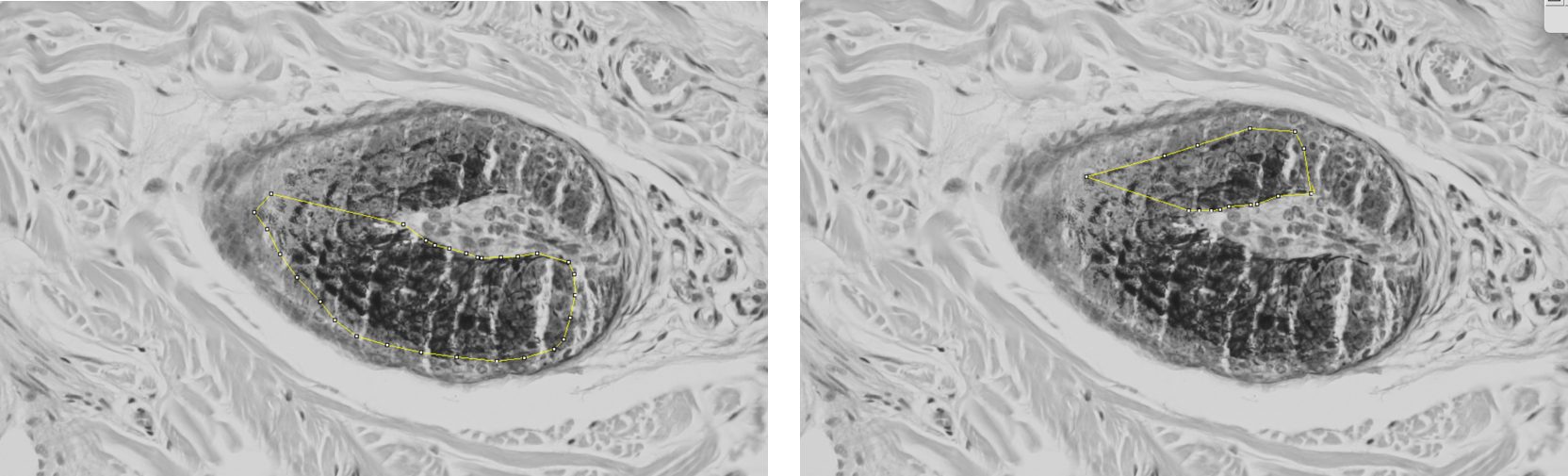

Supplement: Supplementary file 1 [file animals-16-00297-s001.zip › Figure S4/HE Staining Results/BIC/BIC HE.png]

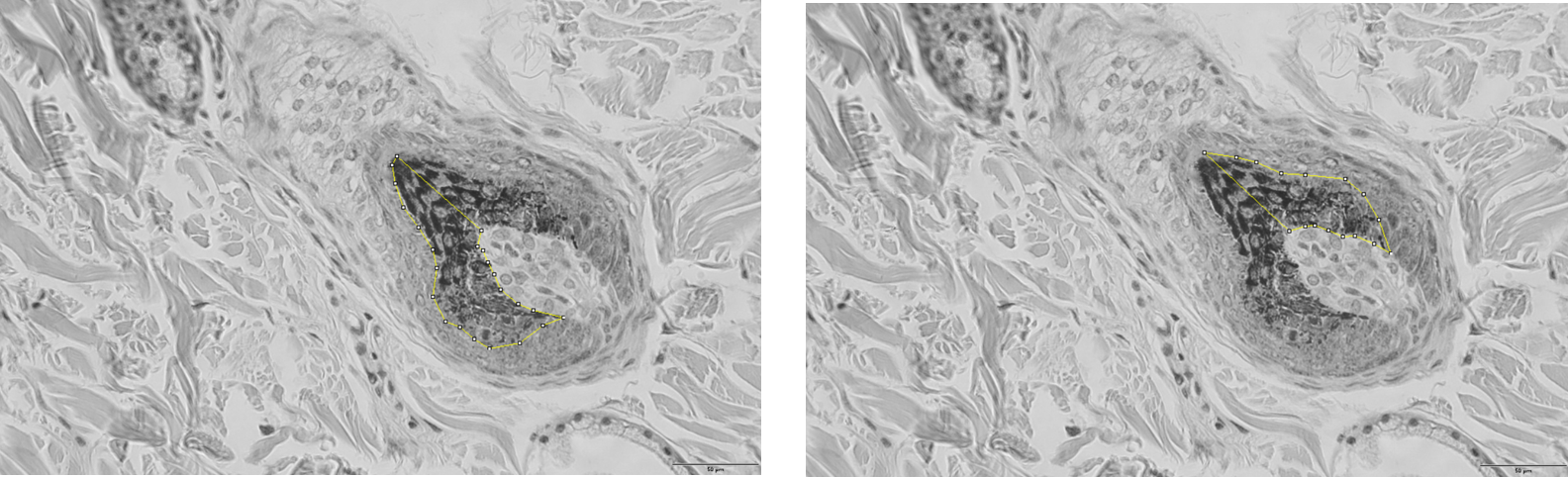

Supplement: Supplementary file 1 [file animals-16-00297-s001.zip › Figure S4/HE Staining Results/BIDC/BIDC HE.png]

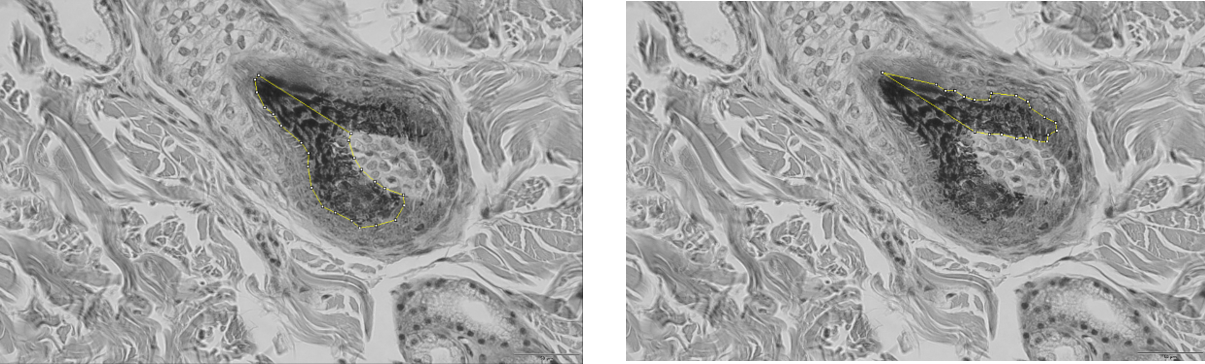

Supplement: Supplementary file 1 [file animals-16-00297-s001.zip › Figure S4/HE Staining Results/BIDM/BIDM HE.png]

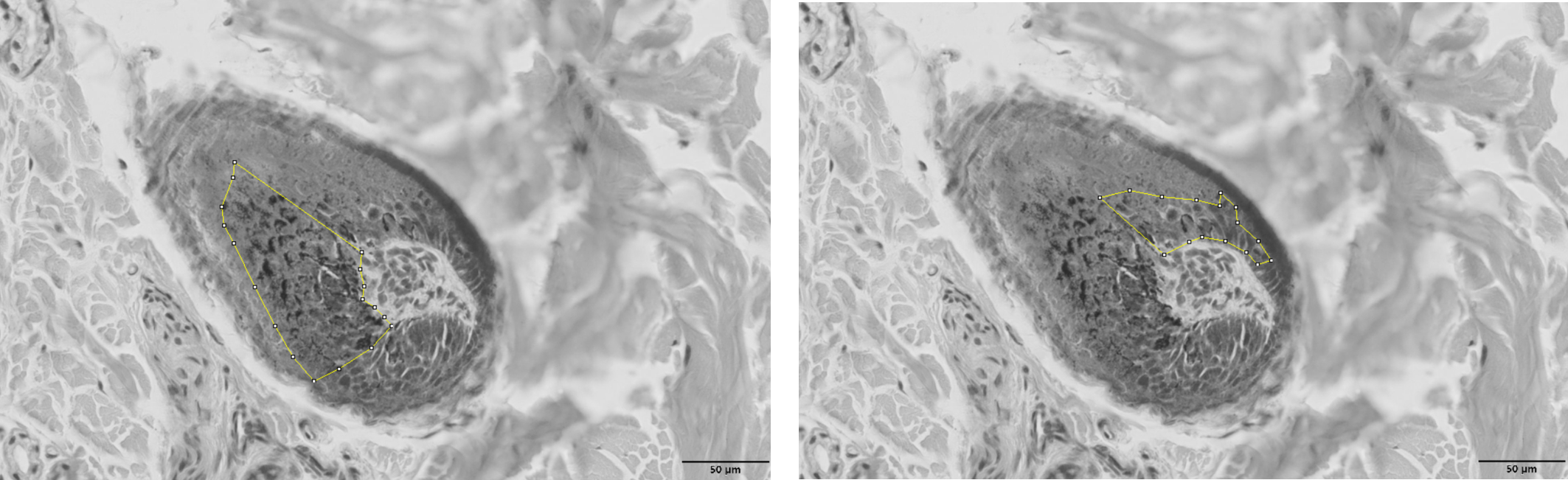

Supplement: Supplementary file 1 [file animals-16-00297-s001.zip › Figure S4/HE Staining Results/BILC/BILC HE.png]

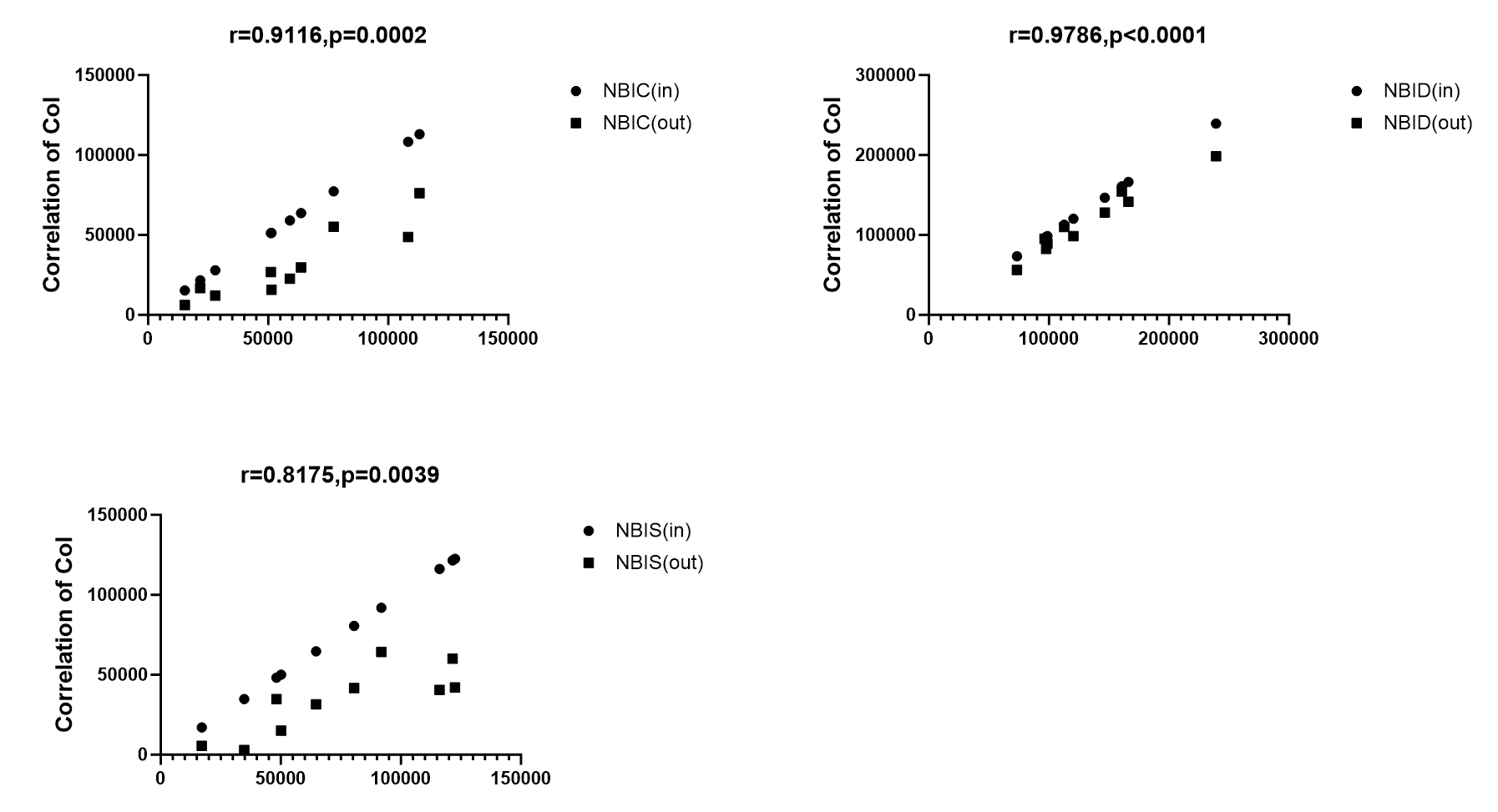

Supplement: Supplementary file 1 [file animals-16-00297-s001.zip › Figure S4/HE Staining Results/NBI pigment deposition symmetry.png]

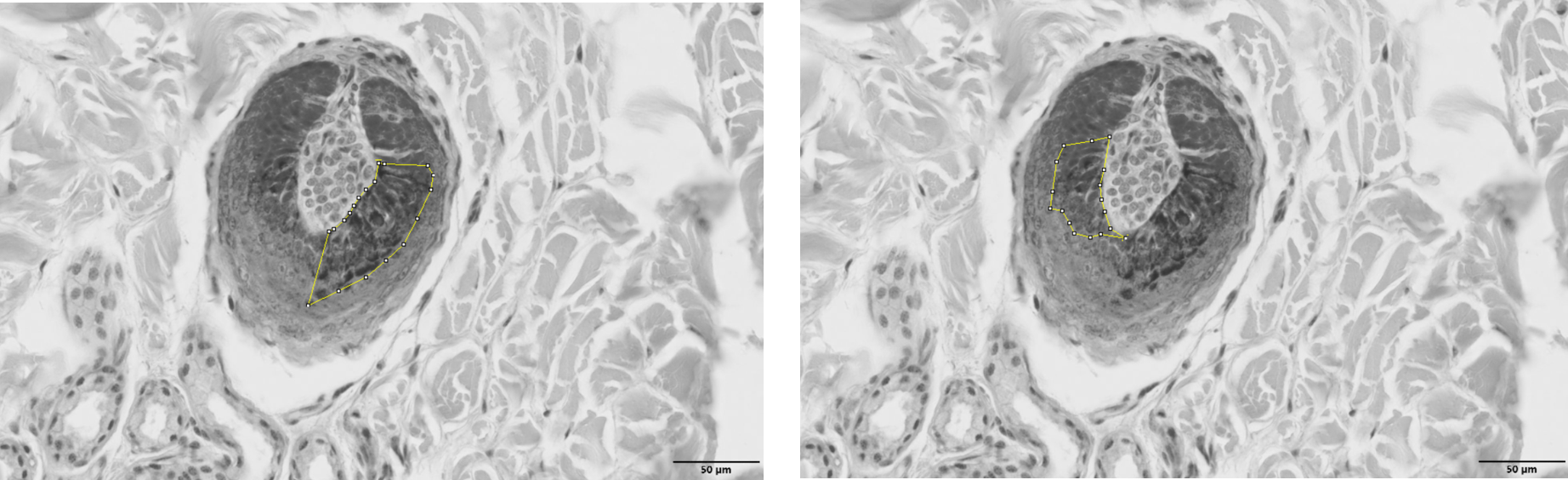

Supplement: Supplementary file 1 [file animals-16-00297-s001.zip › Figure S4/HE Staining Results/NBIC/NBIC HE.png]

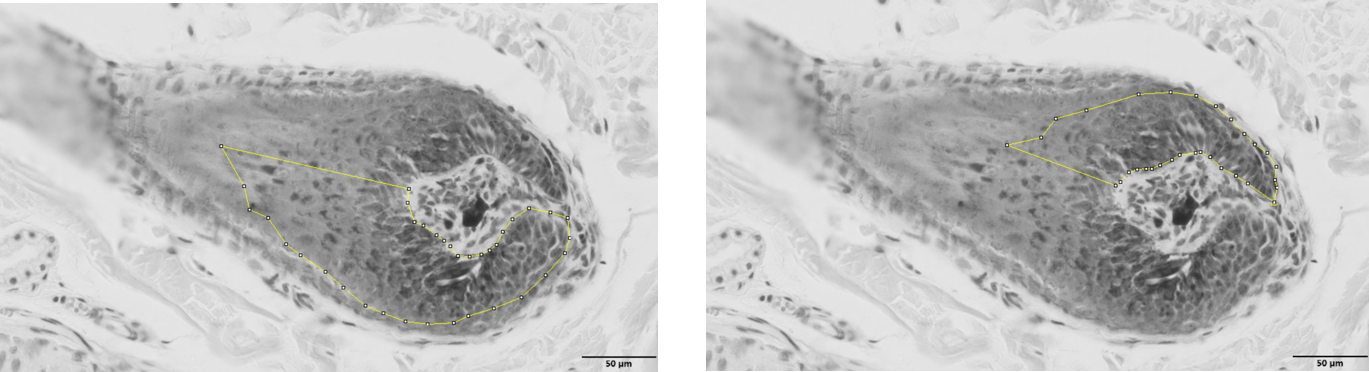

Supplement: Supplementary file 1 [file animals-16-00297-s001.zip › Figure S4/HE Staining Results/NBID/NBID HE.png]

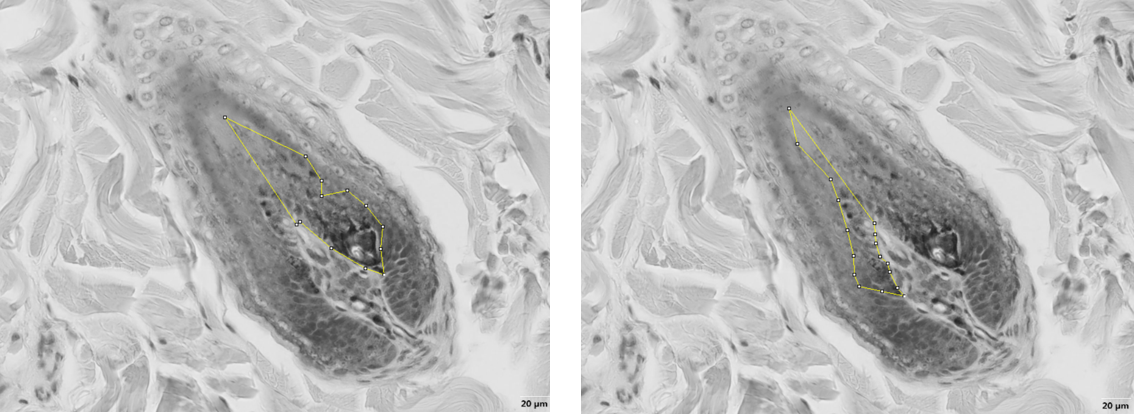

Supplement: Supplementary file 1 [file animals-16-00297-s001.zip › Figure S4/HE Staining Results/NBIS/NBIS HE.png]

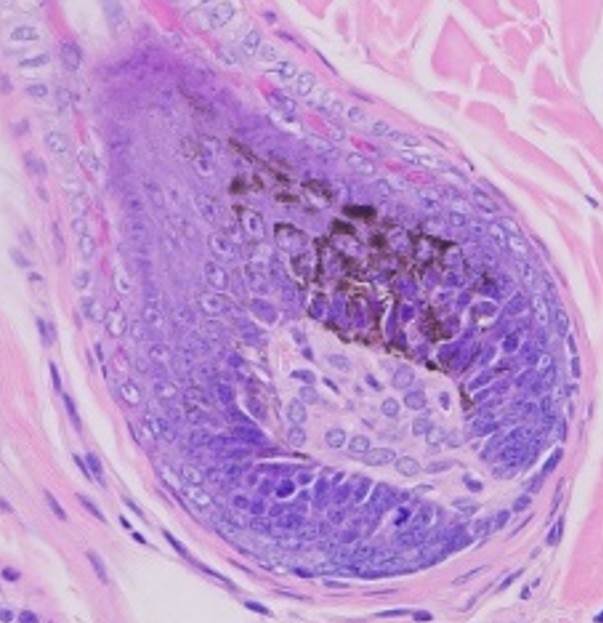

Supplement: Supplementary file 1 [file animals-16-00297-s001.zip › Figure S4/YBC.jpg]

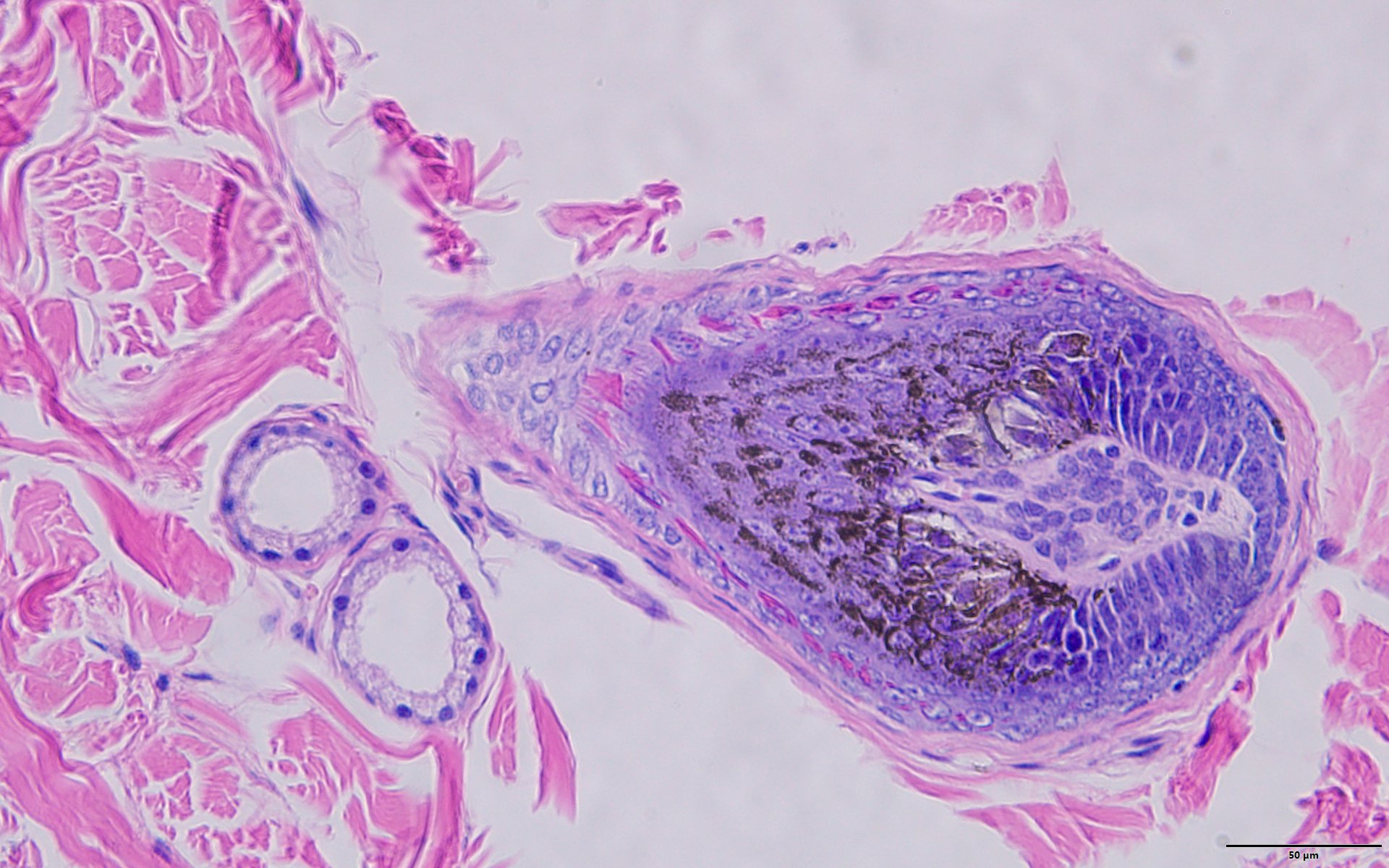

Supplement: Supplementary file 1 [file animals-16-00297-s001.zip › Figure S4/YBD.jpg]

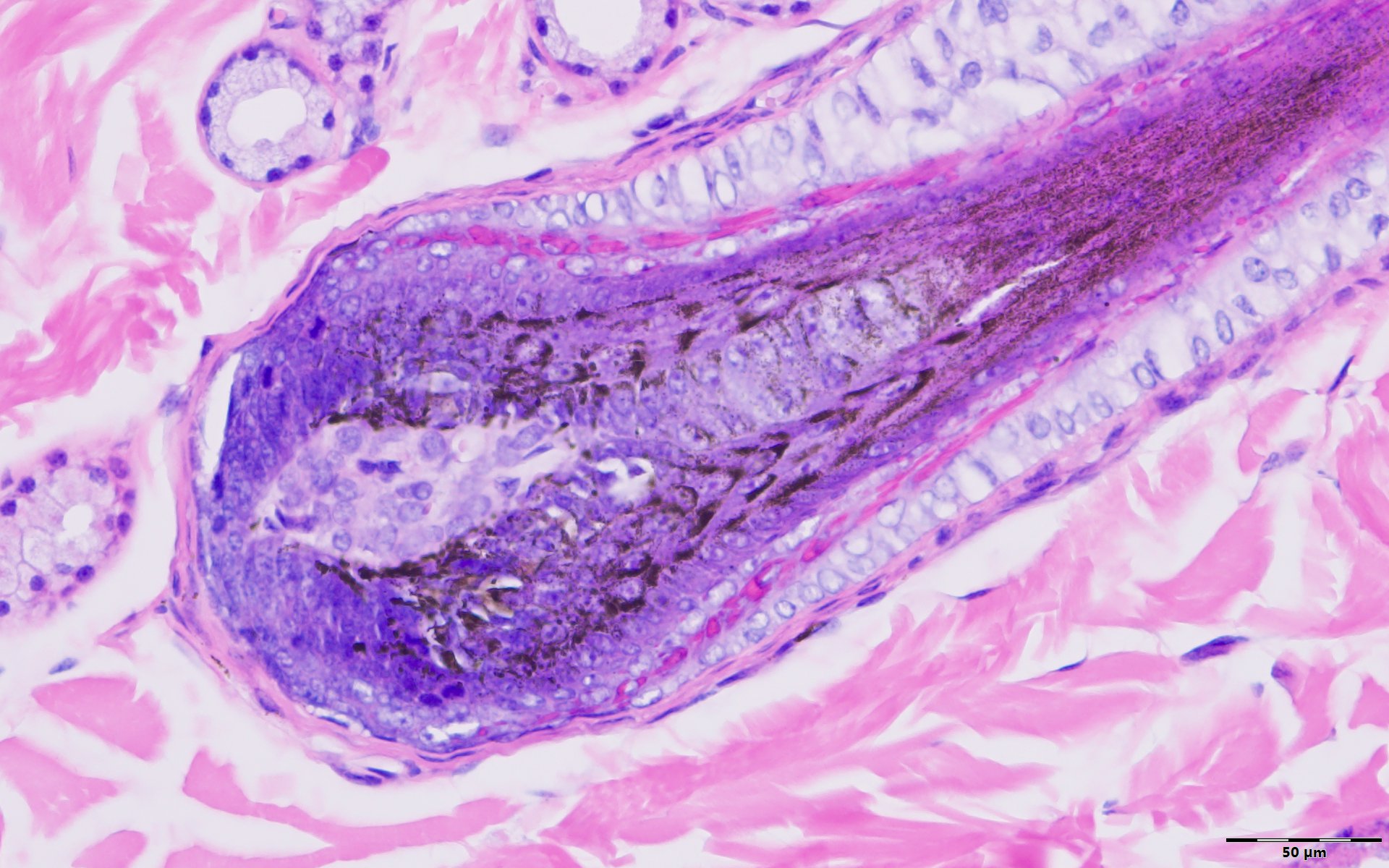

Supplement: Supplementary file 1 [file animals-16-00297-s001.zip › Figure S4/YBDC.jpg]

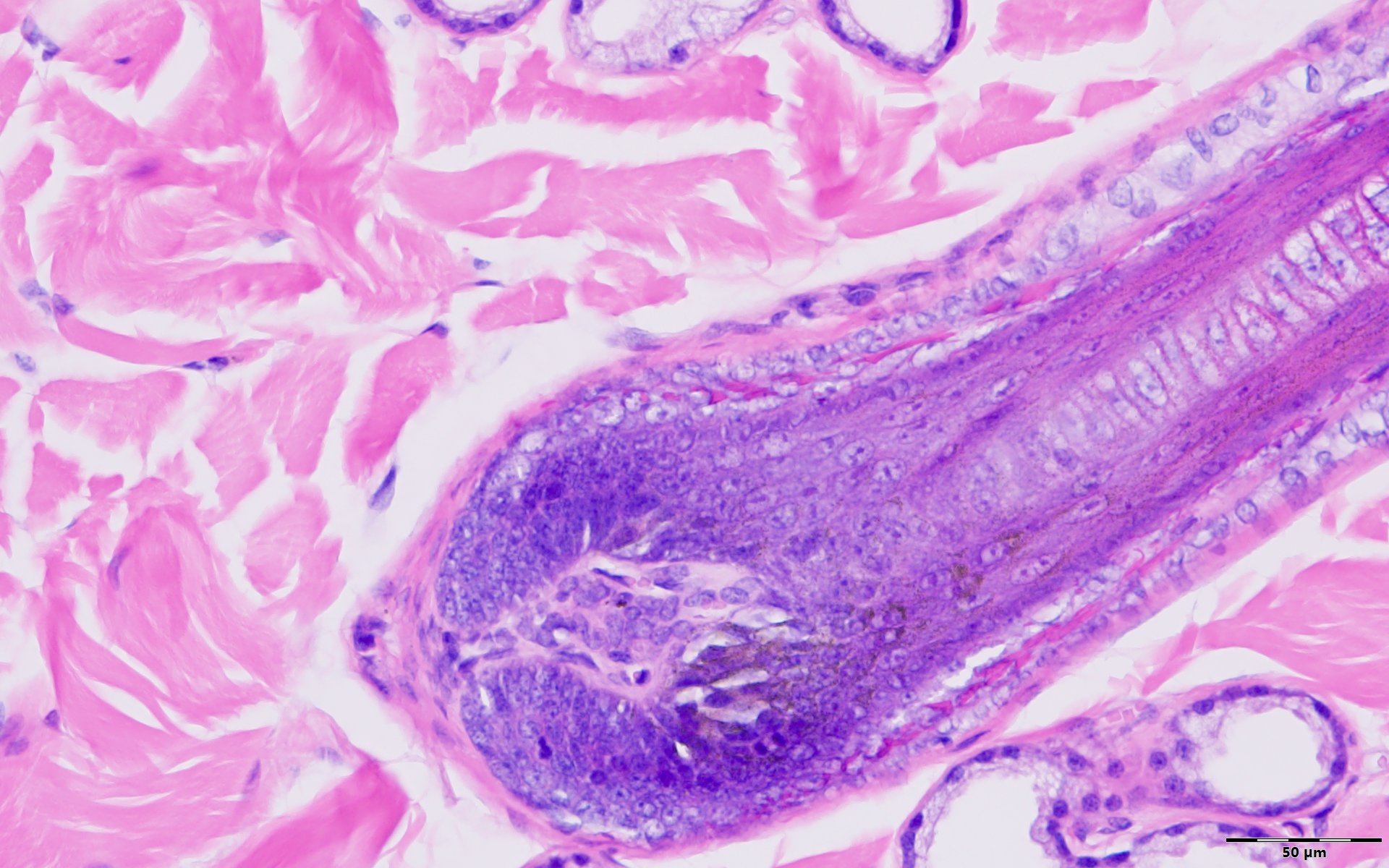

Supplement: Supplementary file 1 [file animals-16-00297-s001.zip › Figure S4/YBLC.jpg]

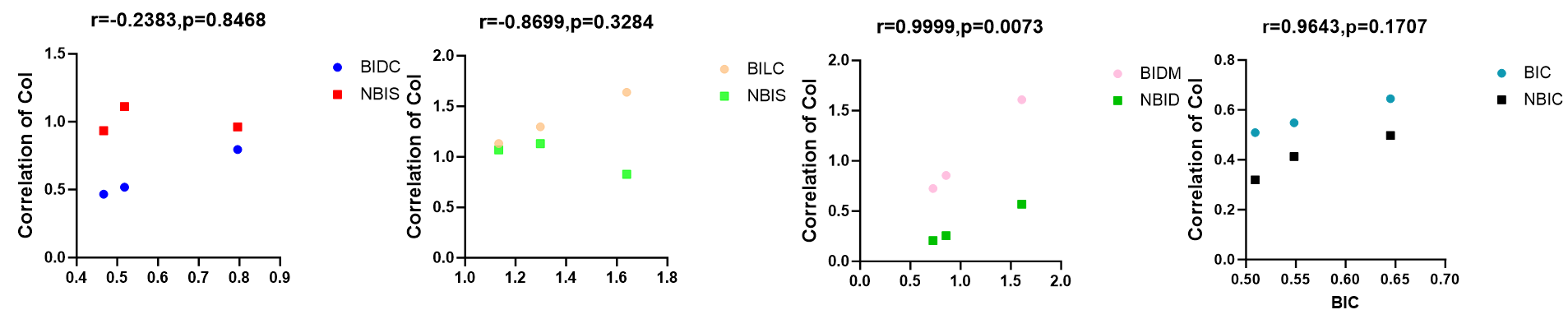

Supplement: Supplementary file 1 [file animals-16-00297-s001.zip › Figure S5/TBX3 expression level (mRNA).png]

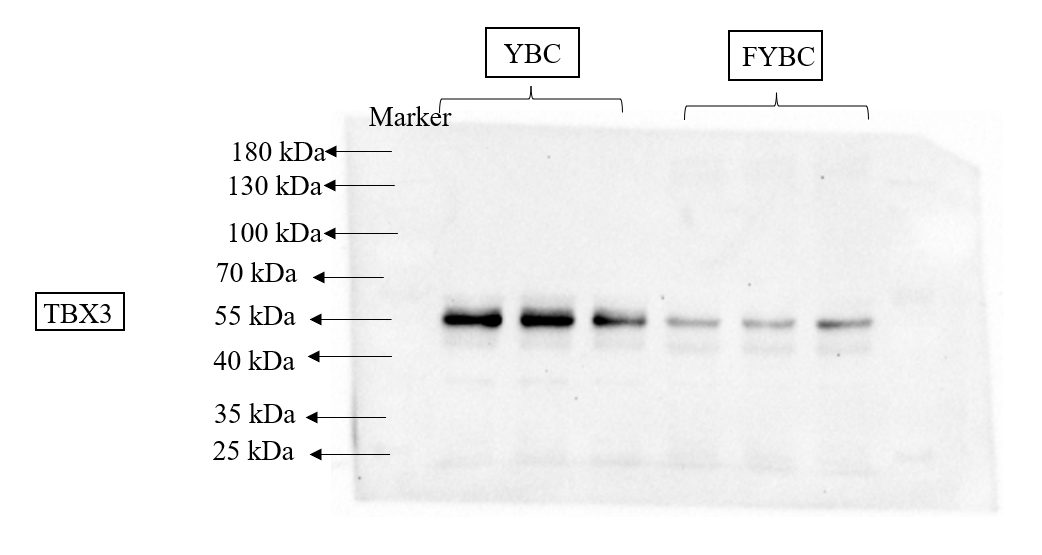

Supplement: Supplementary file 1 [file animals-16-00297-s001.zip › Figure S6/Supplementary documents/YBC FYBC TBX3.png]

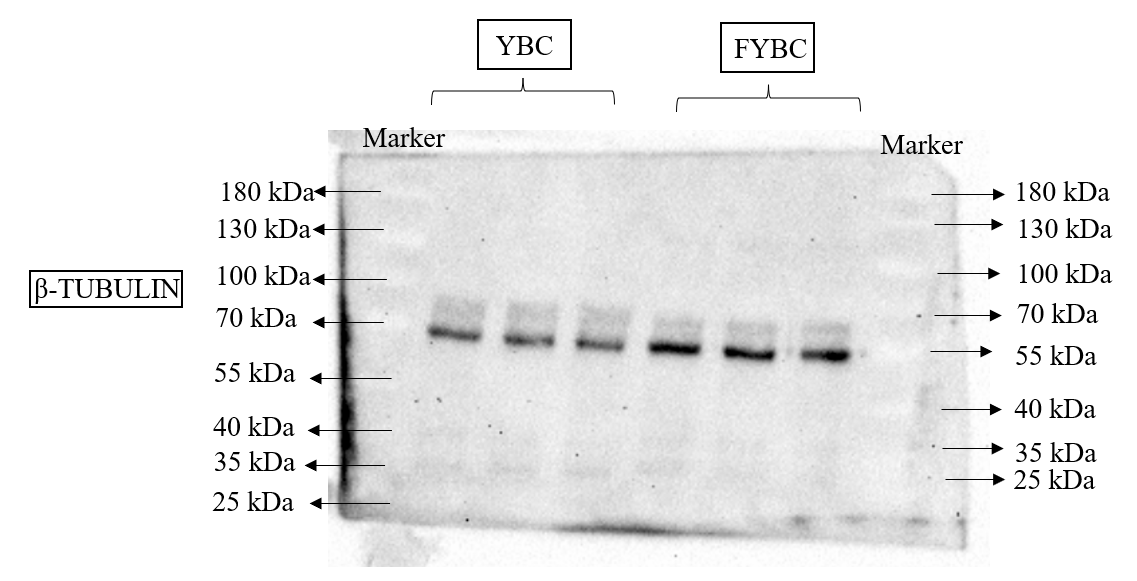

Supplement: Supplementary file 1 [file animals-16-00297-s001.zip › Figure S6/Supplementary documents/YBC FYBC TUBULIN.png]

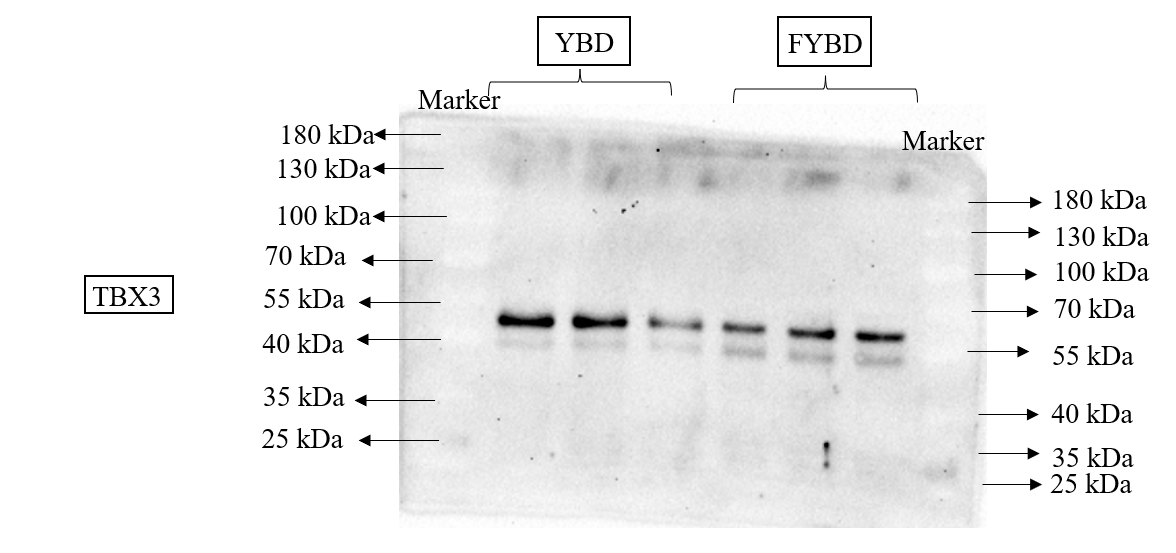

Supplement: Supplementary file 1 [file animals-16-00297-s001.zip › Figure S6/Supplementary documents/YBD FYBD TBX3.png]

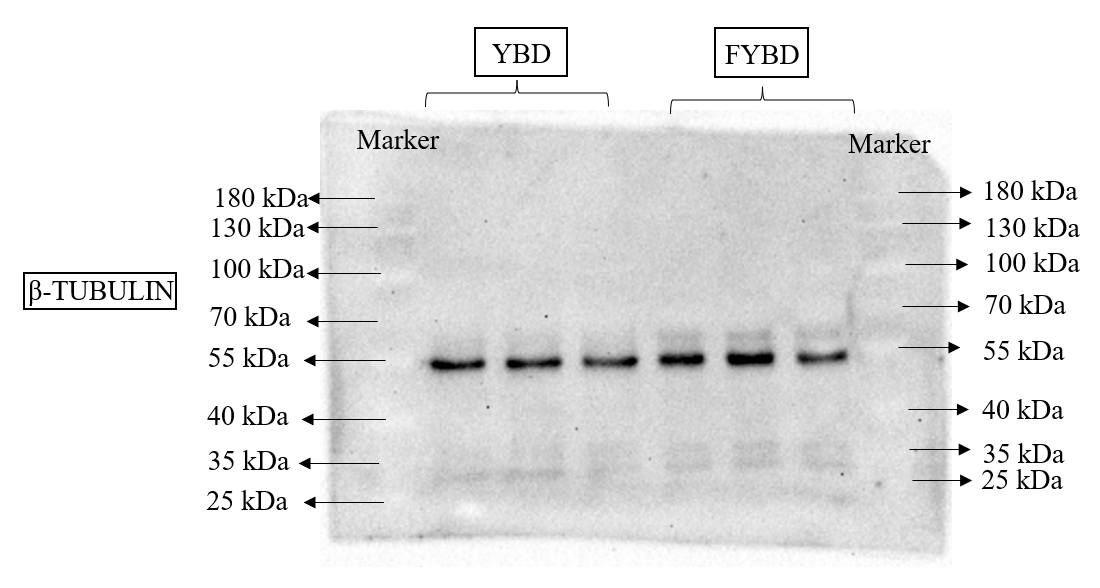

Supplement: Supplementary file 1 [file animals-16-00297-s001.zip › Figure S6/Supplementary documents/YBD FYBD TUBULIN.png]

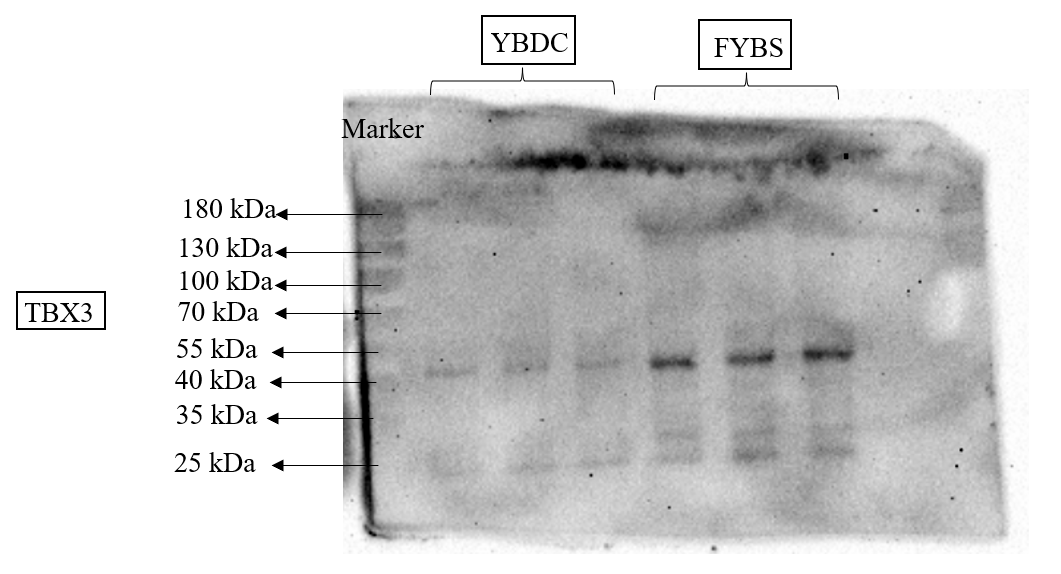

Supplement: Supplementary file 1 [file animals-16-00297-s001.zip › Figure S6/Supplementary documents/YBDC FYBS TBX3.png]

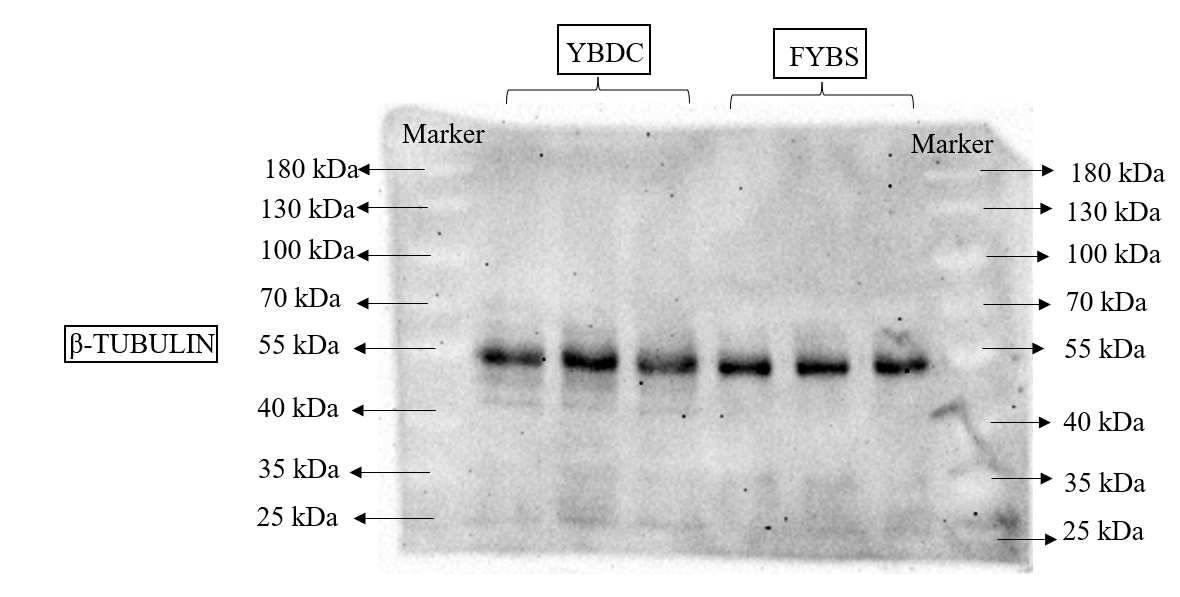

Supplement: Supplementary file 1 [file animals-16-00297-s001.zip › Figure S6/Supplementary documents/YBDC FYBS TUBULIN.png]

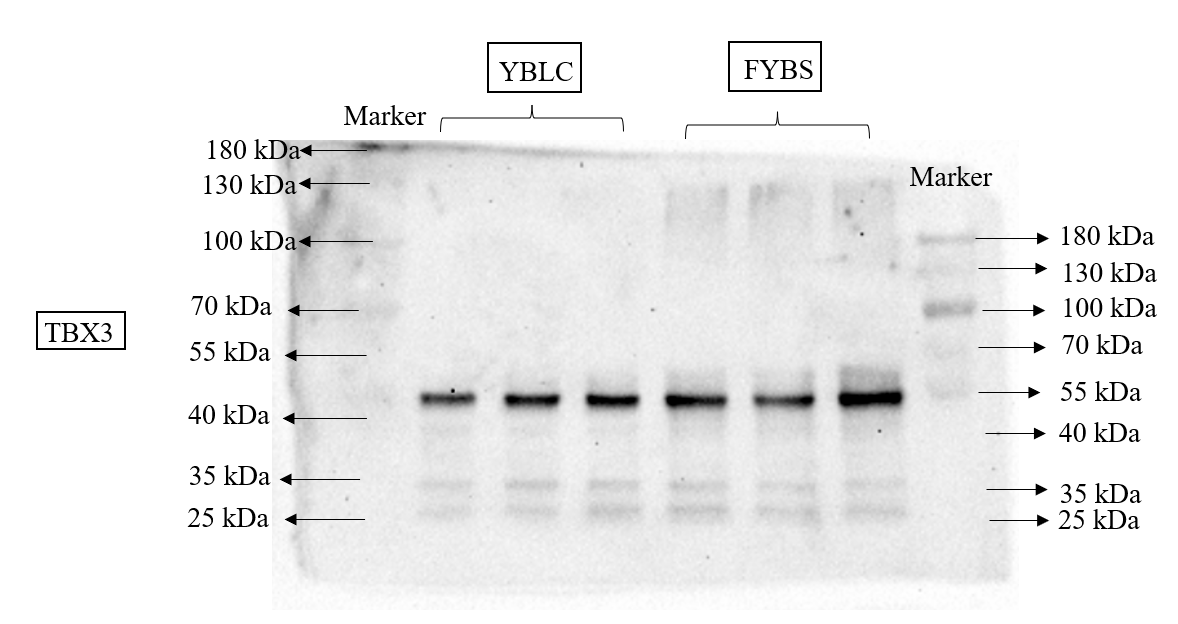

Supplement: Supplementary file 1 [file animals-16-00297-s001.zip › Figure S6/Supplementary documents/YBLC FYBS TBX3.png]

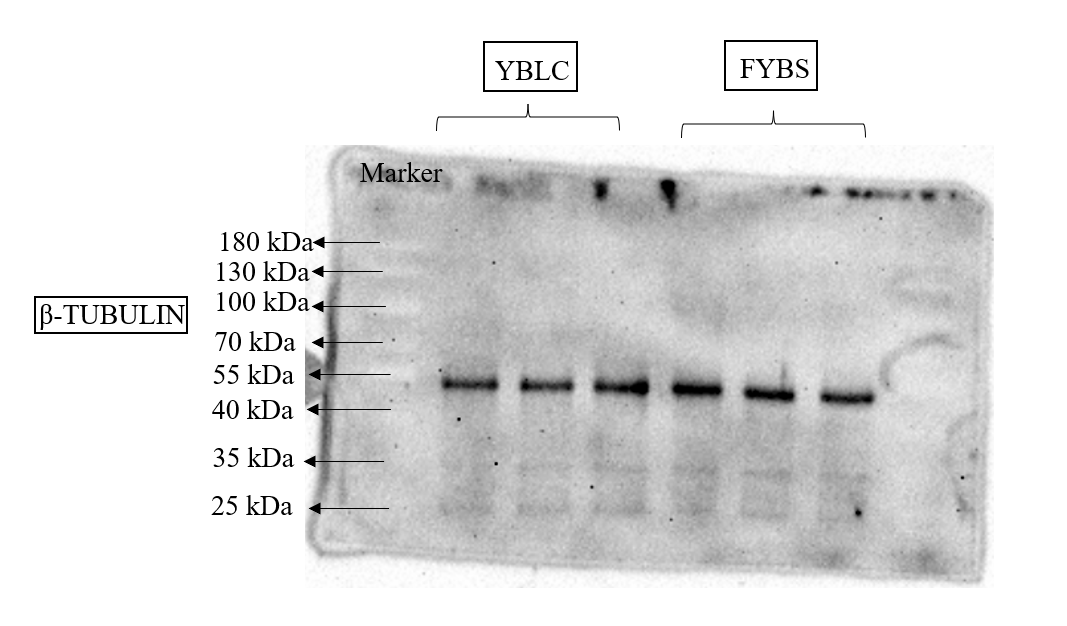

Supplement: Supplementary file 1 [file animals-16-00297-s001.zip › Figure S6/Supplementary documents/YBLC FYBS TUBULIN.png]

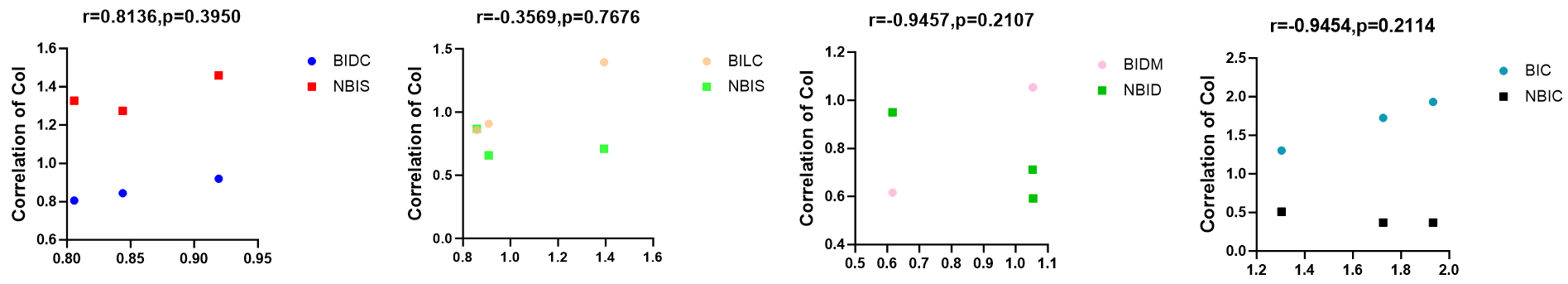

Supplement: Supplementary file 1 [file animals-16-00297-s001.zip › Figure S6/TBX3 expression level (WB).png]

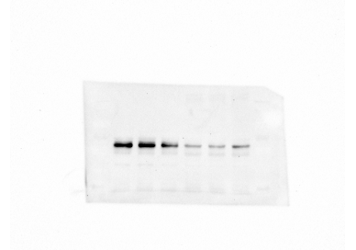

Supplement: Supplementary file 1 [file animals-16-00297-s001.zip › Figure S6/YBC FYBC TBX3.tif]

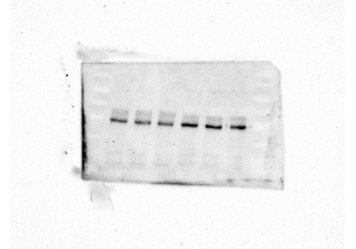

Supplement: Supplementary file 1 [file animals-16-00297-s001.zip › Figure S6/YBC FYBC TUBULIN.tif]

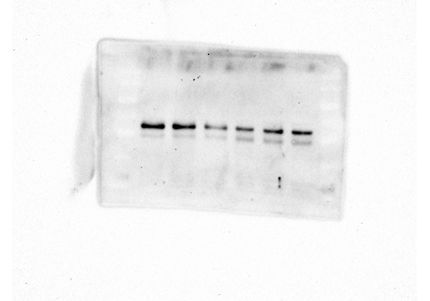

Supplement: Supplementary file 1 [file animals-16-00297-s001.zip › Figure S6/YBD FYBD TBX3.tif]

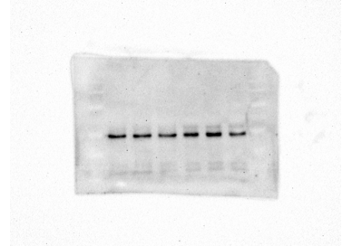

Supplement: Supplementary file 1 [file animals-16-00297-s001.zip › Figure S6/YBD FYBD TYBULIN.tif]

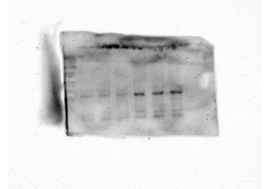

Supplement: Supplementary file 1 [file animals-16-00297-s001.zip › Figure S6/YBDC FYBS TBX3.tif]

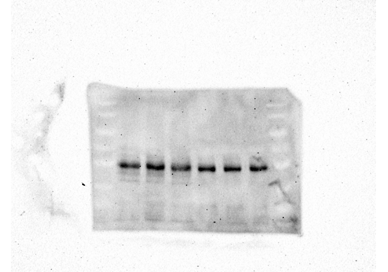

Supplement: Supplementary file 1 [file animals-16-00297-s001.zip › Figure S6/YBDC FYBS TUBULIN.tif]

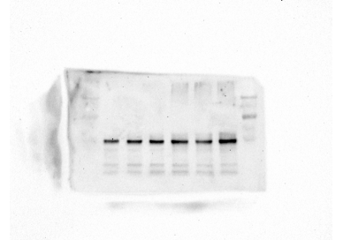

Supplement: Supplementary file 1 [file animals-16-00297-s001.zip › Figure S6/YBLC FYBS TBX3'.tif]

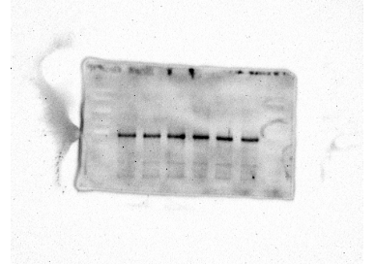

Supplement: Supplementary file 1 [file animals-16-00297-s001.zip › Figure S6/YBLC FYBS TUBULIN.tif]

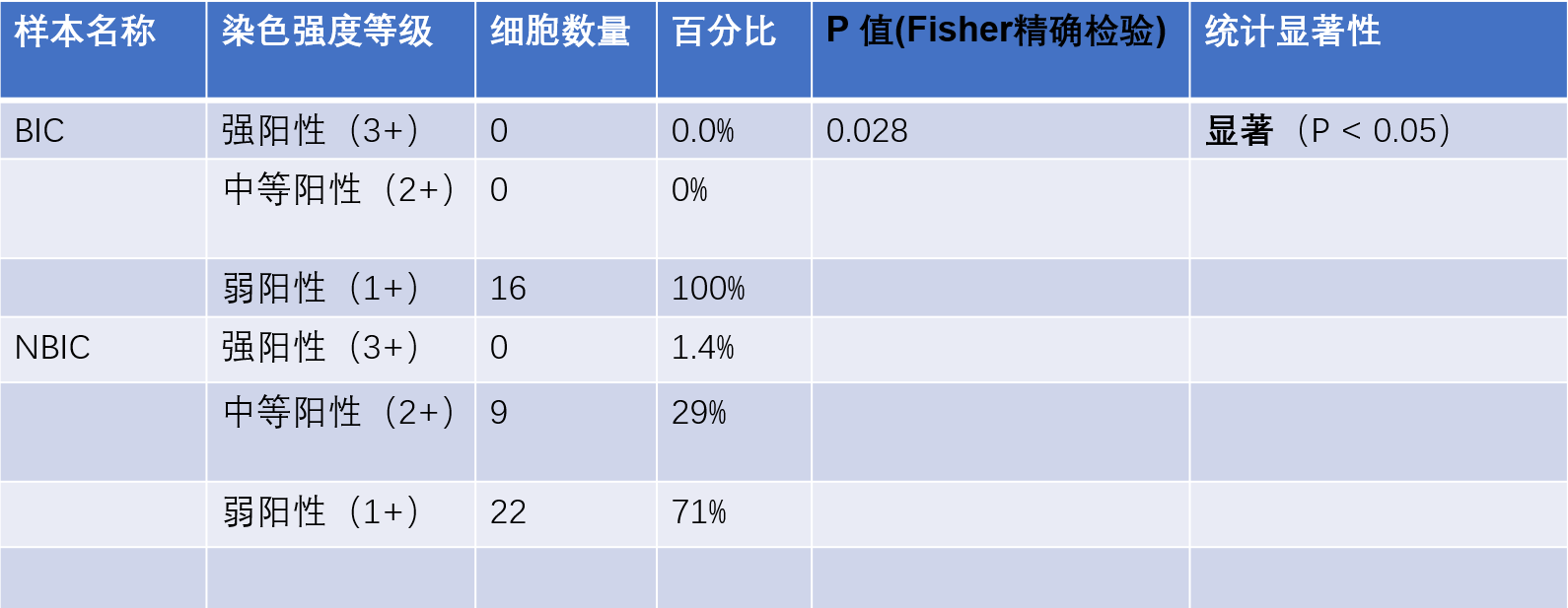

Supplement: Supplementary file 1 [file animals-16-00297-s001.zip › Figure S7/epidermis/epidermis/results/BIC VS NBIC.png]

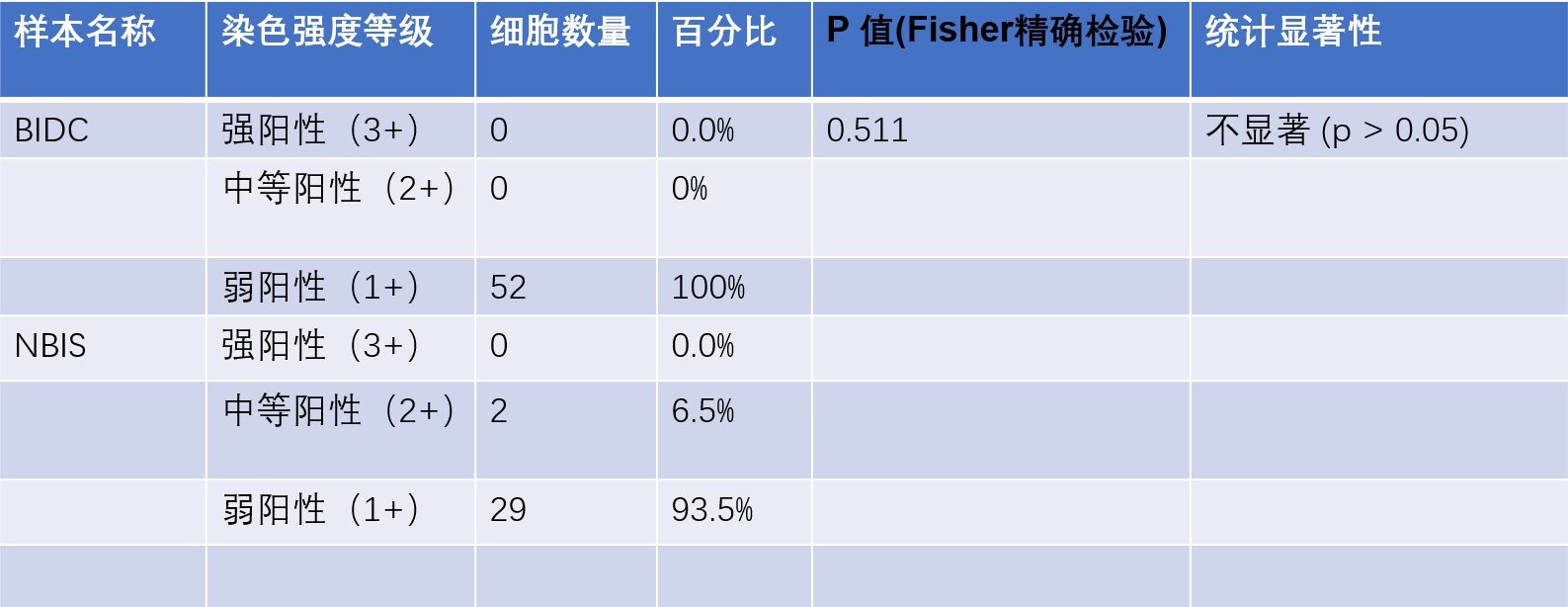

Supplement: Supplementary file 1 [file animals-16-00297-s001.zip › Figure S7/epidermis/epidermis/results/BIDC VS NBIS.png]

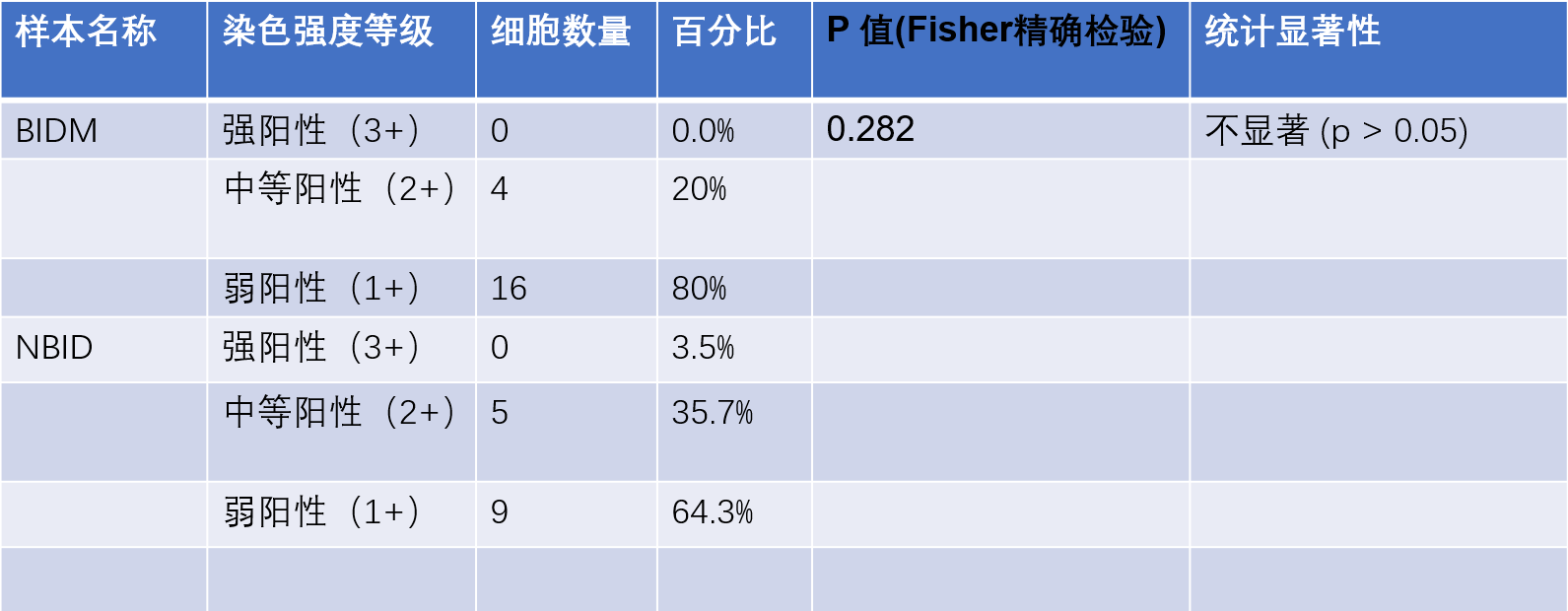

Supplement: Supplementary file 1 [file animals-16-00297-s001.zip › Figure S7/epidermis/epidermis/results/BIDM VS NBID.png]

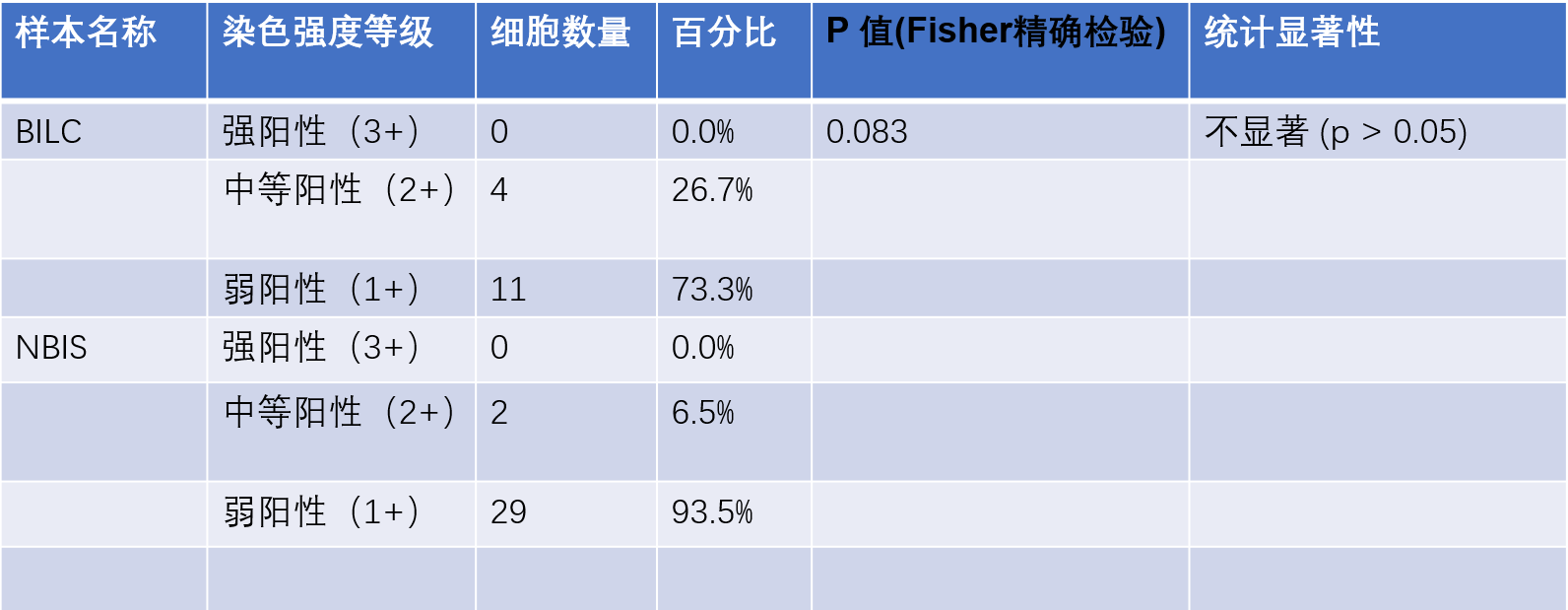

Supplement: Supplementary file 1 [file animals-16-00297-s001.zip › Figure S7/epidermis/epidermis/results/BILC VS NBIS.png]

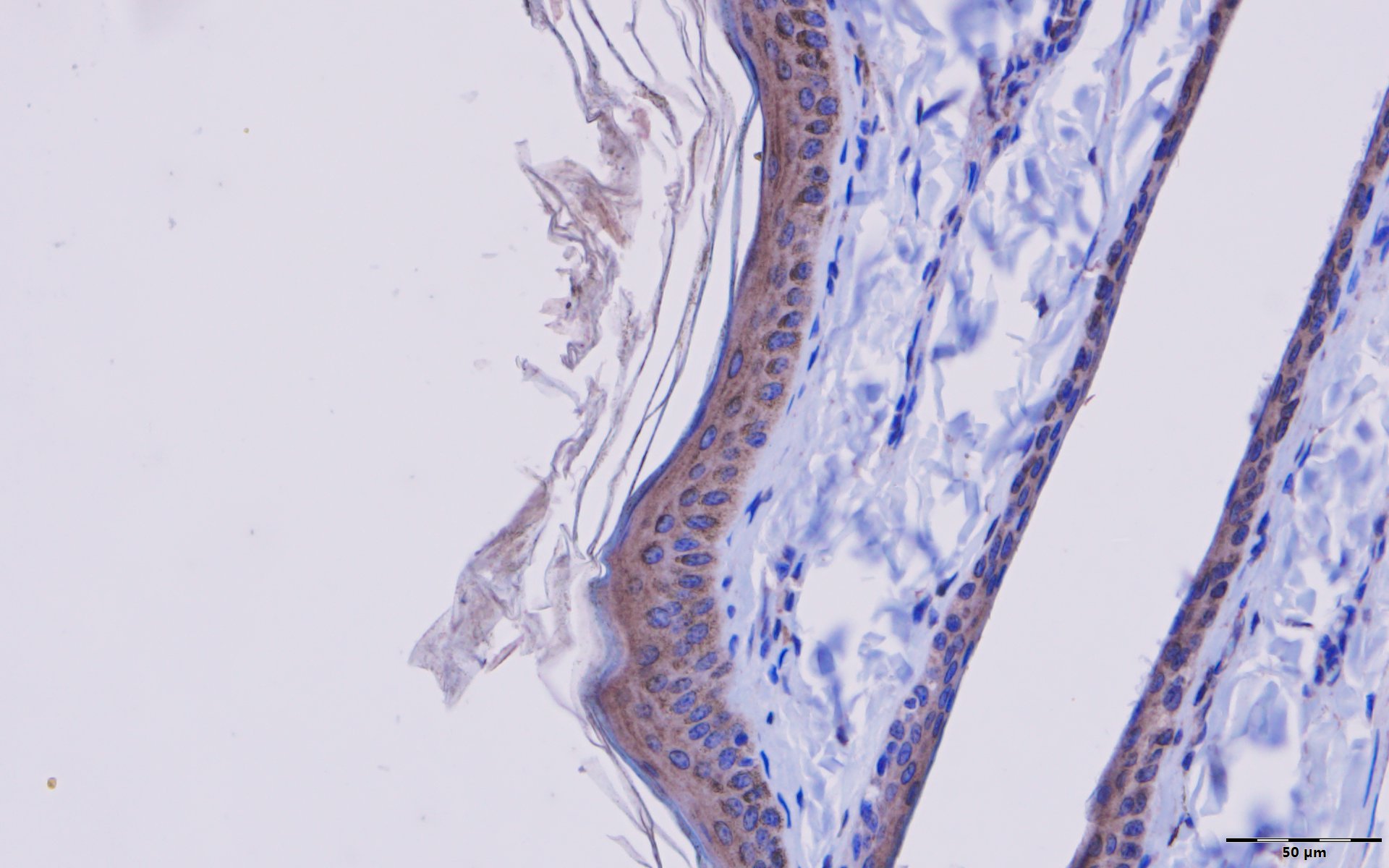

Supplement: Supplementary file 1 [file animals-16-00297-s001.zip › Figure S7/epidermis/FYBC.jpg]

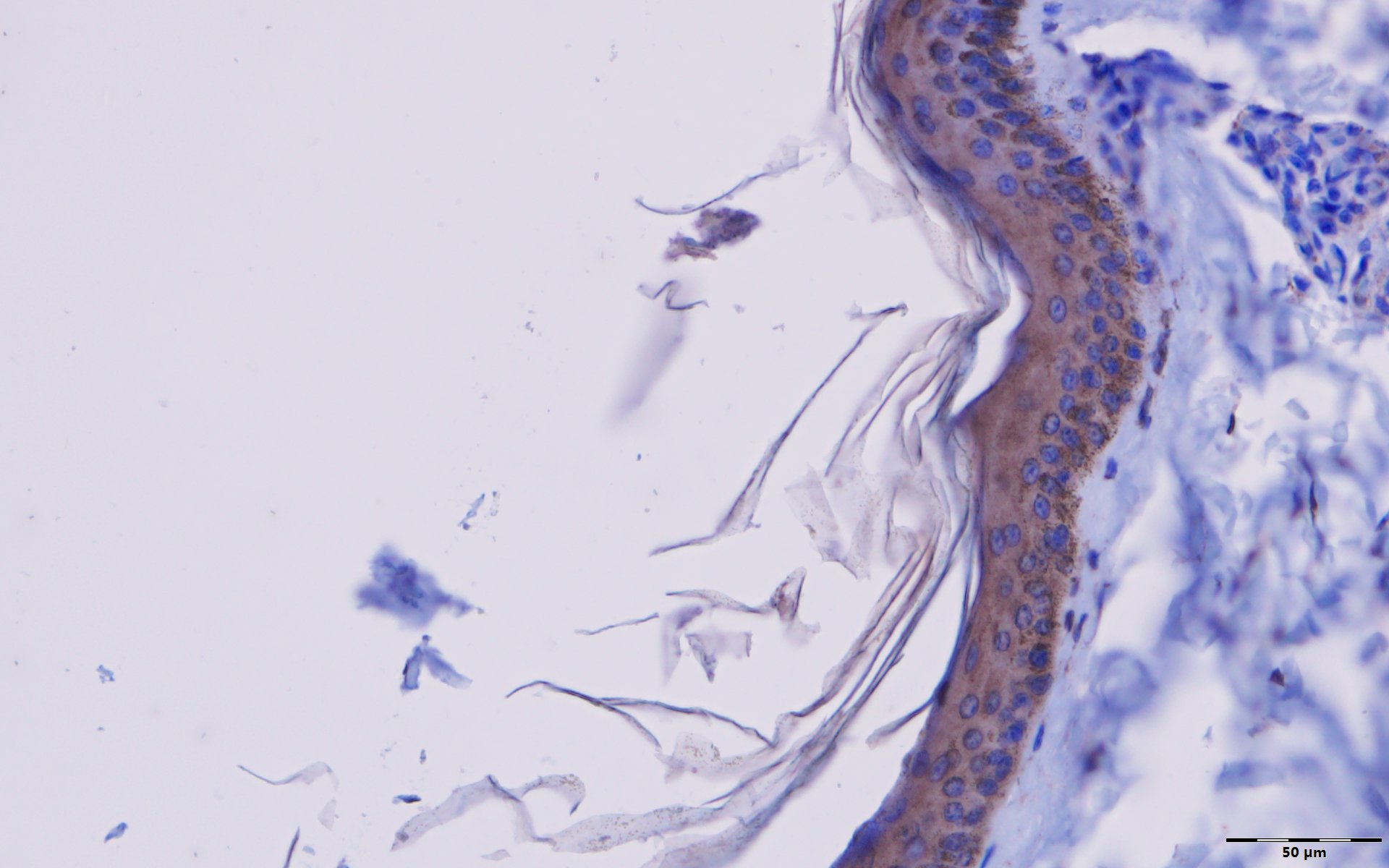

Supplement: Supplementary file 1 [file animals-16-00297-s001.zip › Figure S7/epidermis/FYBD.jpg]

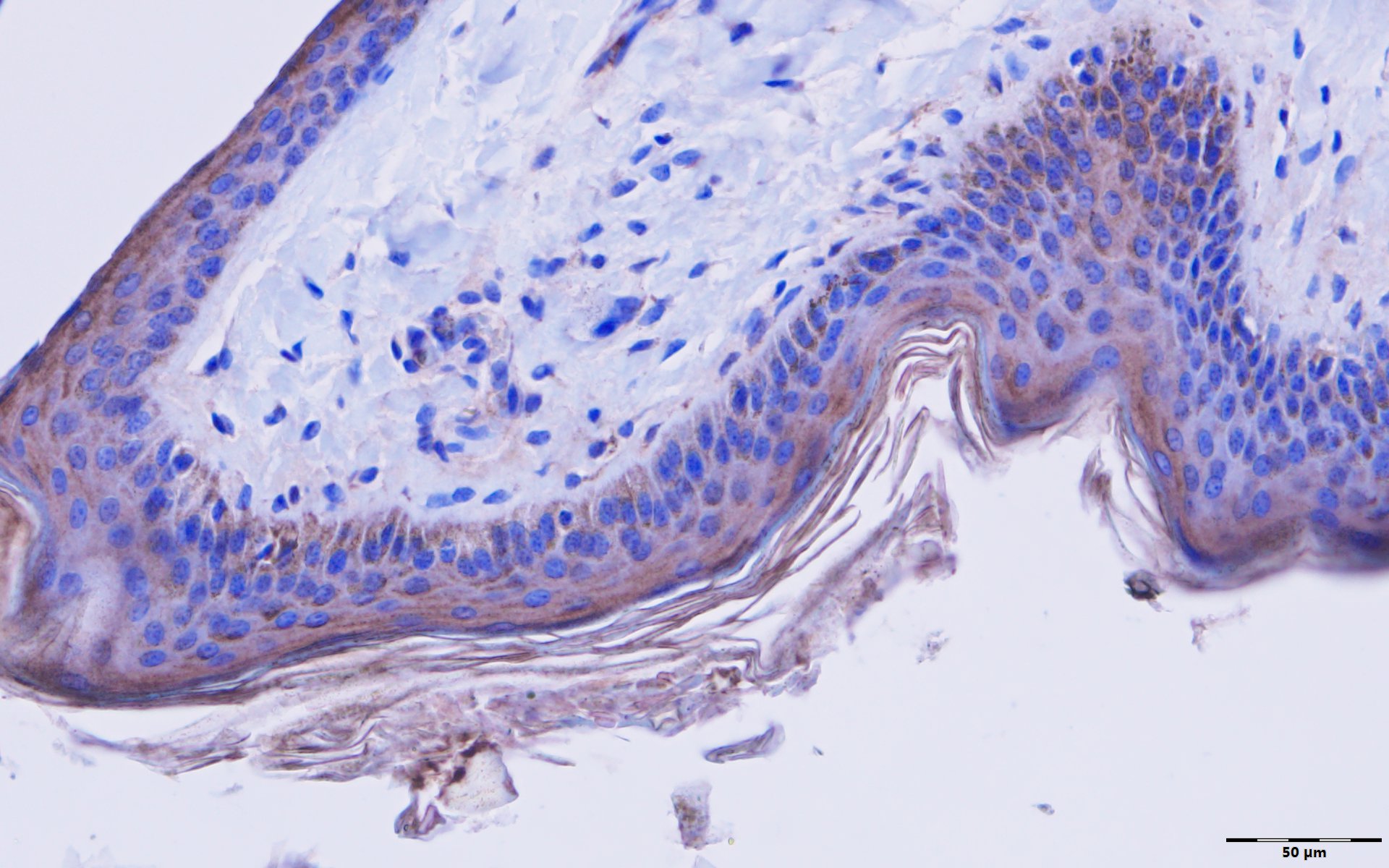

Supplement: Supplementary file 1 [file animals-16-00297-s001.zip › Figure S7/epidermis/FYBS.jpg]

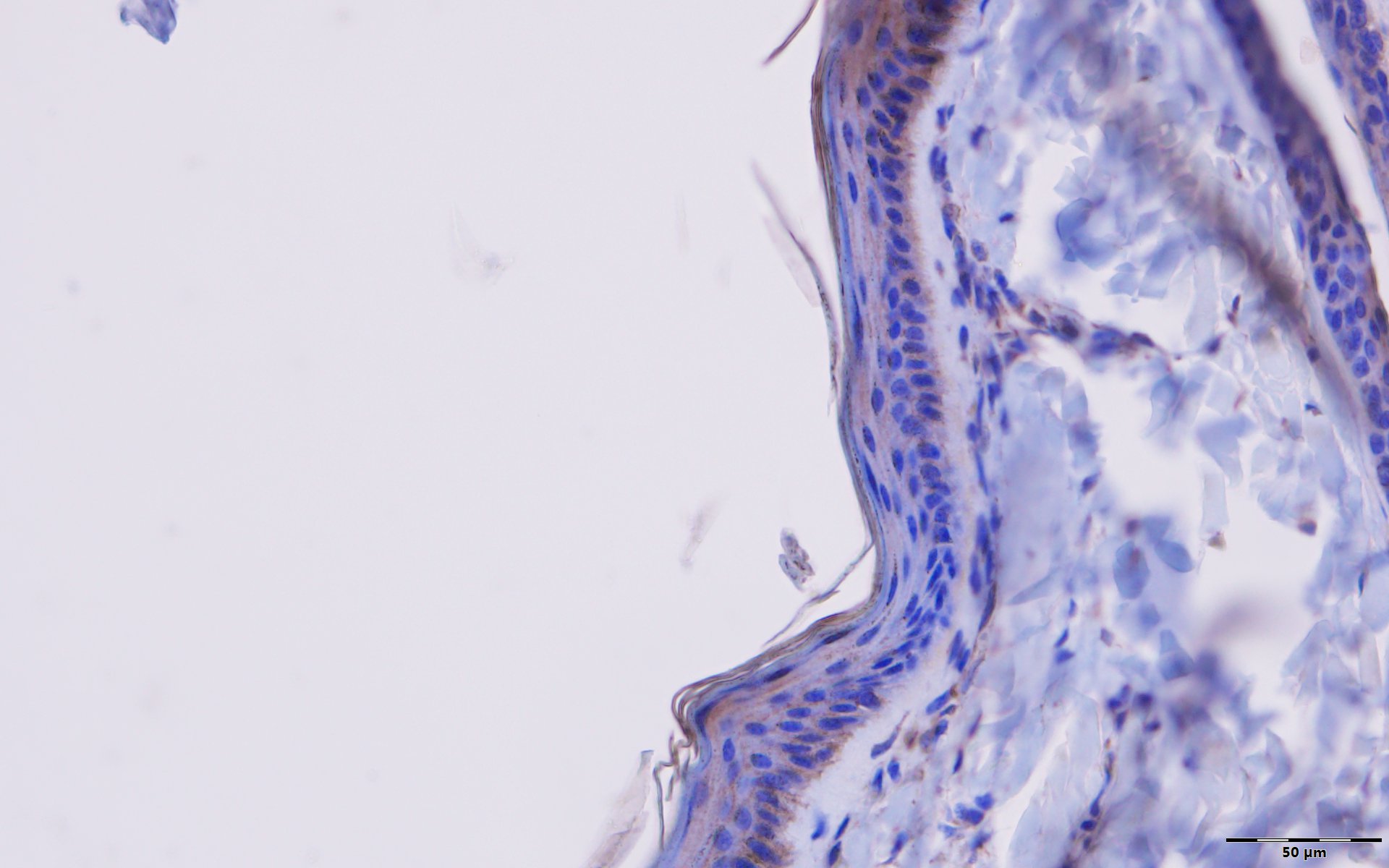

Supplement: Supplementary file 1 [file animals-16-00297-s001.zip › Figure S7/epidermis/YBC.jpg]

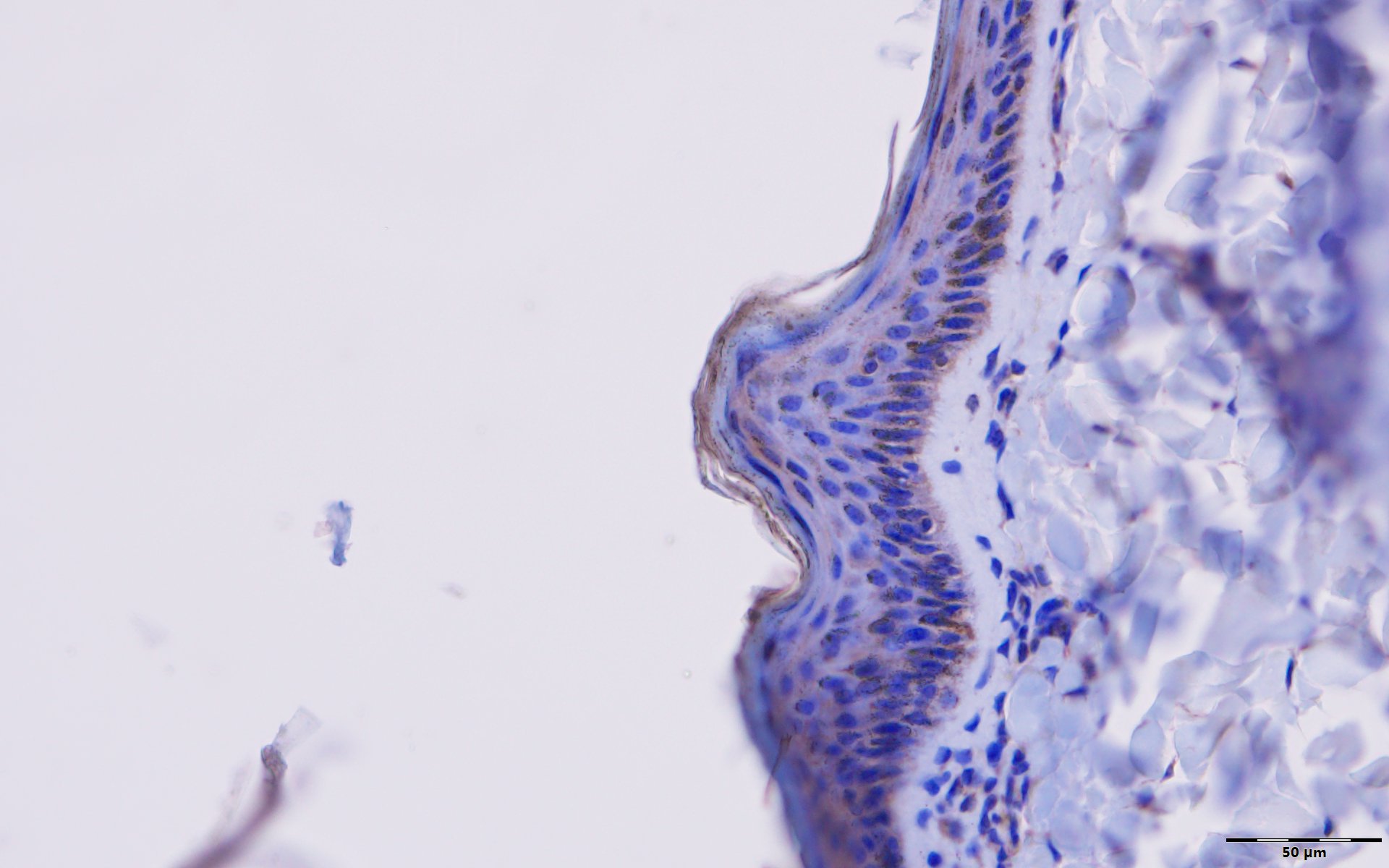

Supplement: Supplementary file 1 [file animals-16-00297-s001.zip › Figure S7/epidermis/YBD.jpg]

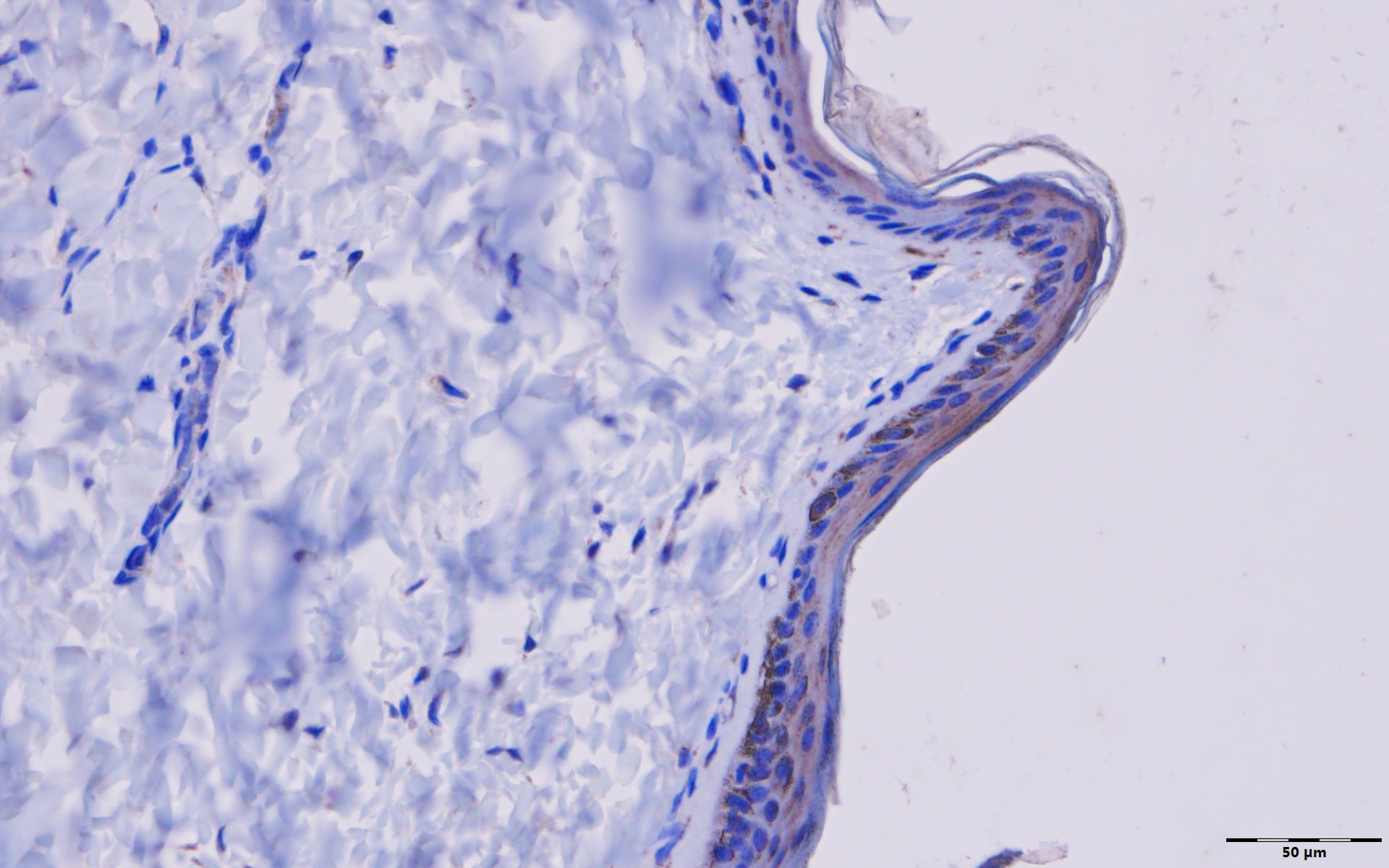

Supplement: Supplementary file 1 [file animals-16-00297-s001.zip › Figure S7/epidermis/YBDC.jpg]

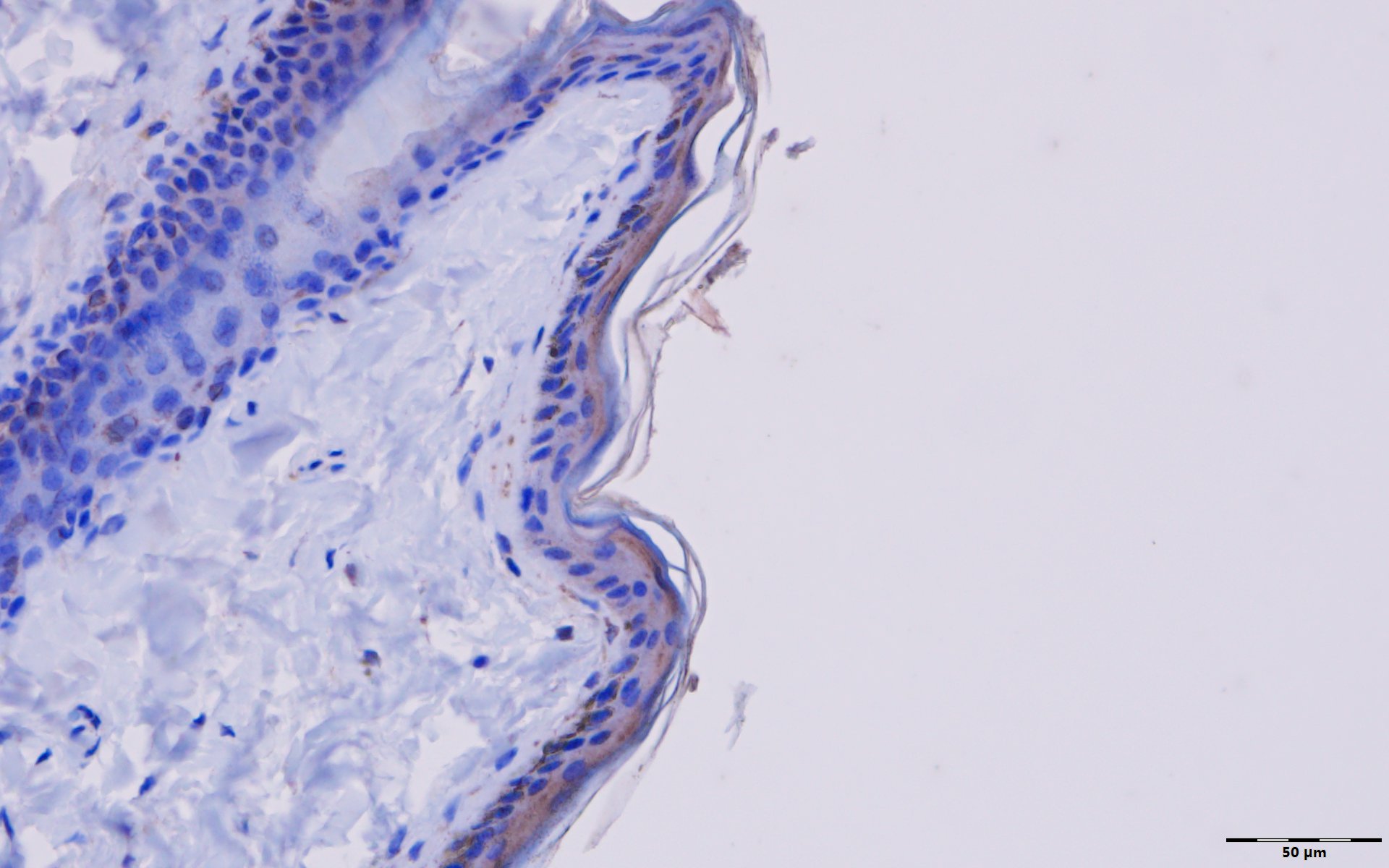

Supplement: Supplementary file 1 [file animals-16-00297-s001.zip › Figure S7/epidermis/YBLC.jpg]

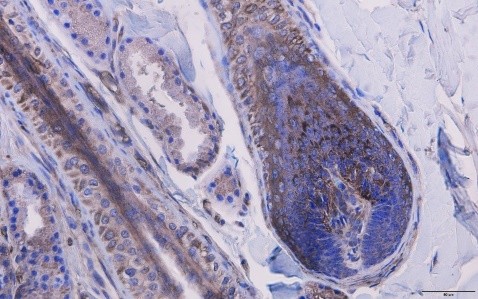

Supplement: Supplementary file 1 [file animals-16-00297-s001.zip › Figure S7/hair bulb/FYBC.jpg]

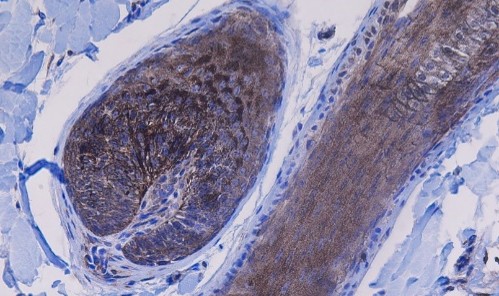

Supplement: Supplementary file 1 [file animals-16-00297-s001.zip › Figure S7/hair bulb/FYBD.jpg]

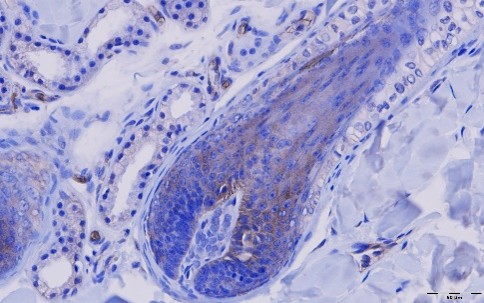

Supplement: Supplementary file 1 [file animals-16-00297-s001.zip › Figure S7/hair bulb/FYBS.jpg]

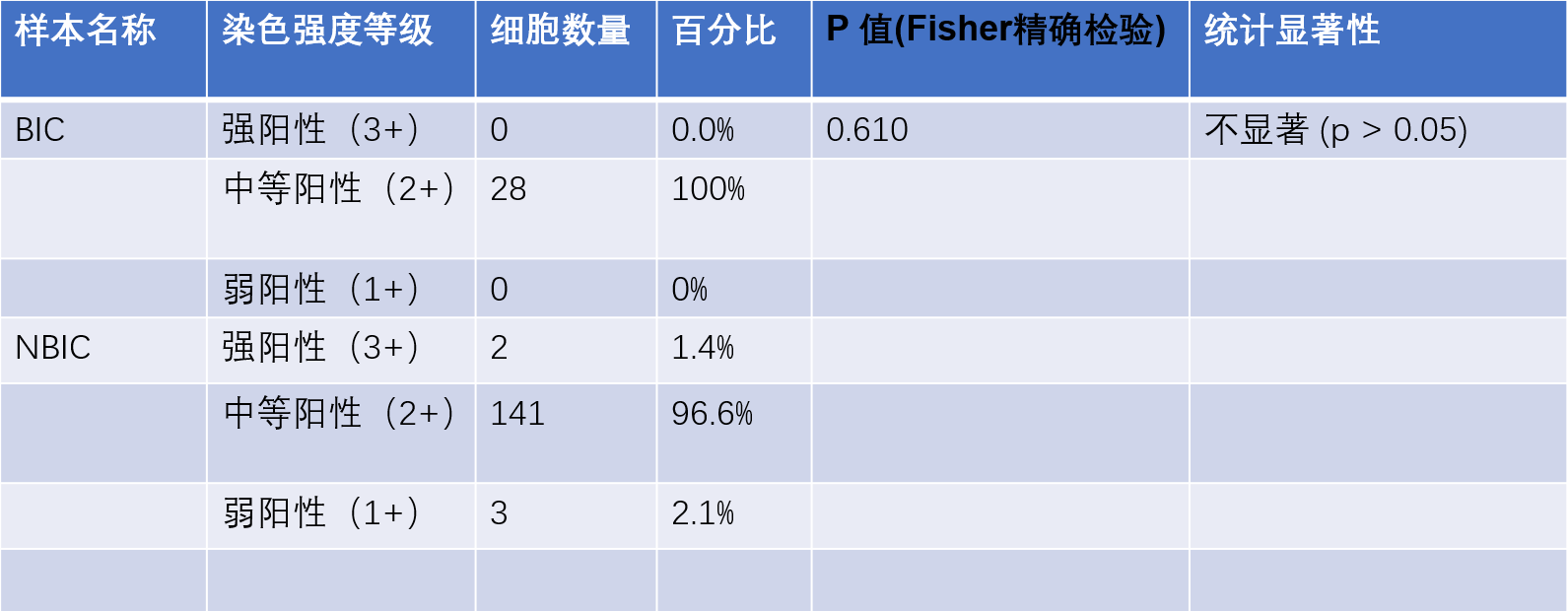

Supplement: Supplementary file 1 [file animals-16-00297-s001.zip › Figure S7/hair bulb/hair bulb/results/BIC VS NBIC.png]

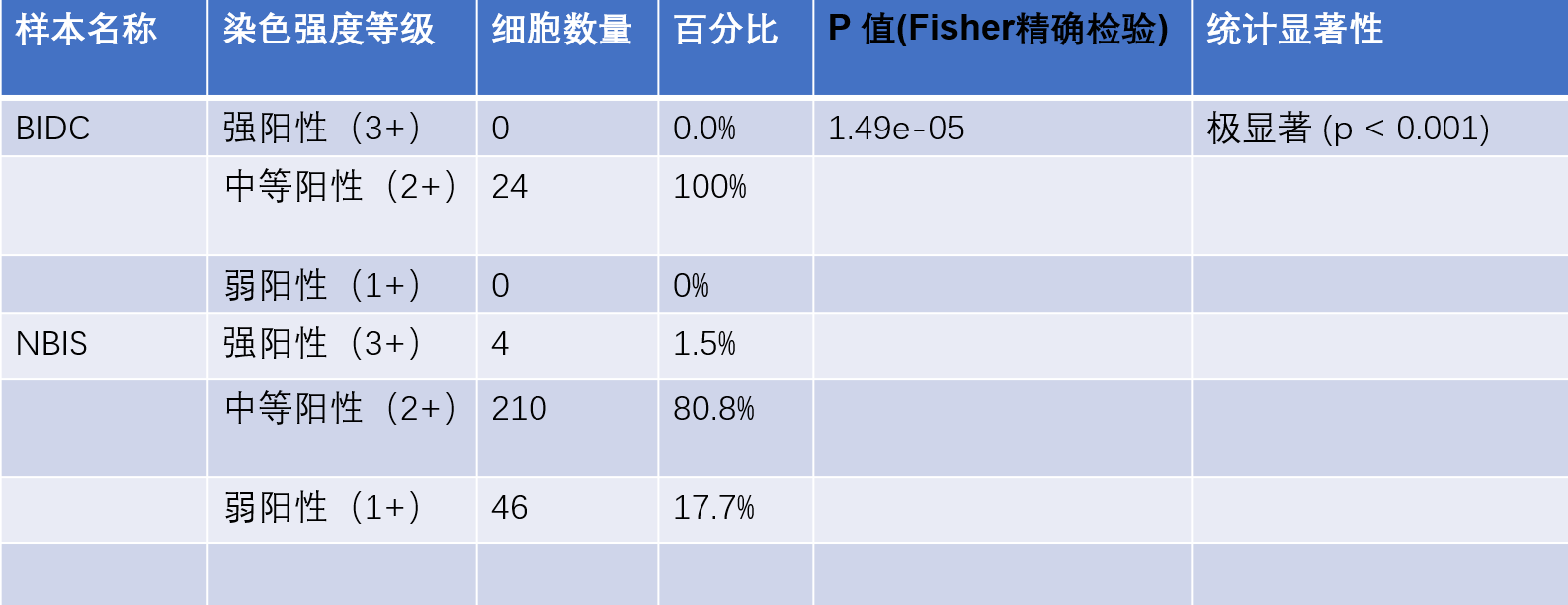

Supplement: Supplementary file 1 [file animals-16-00297-s001.zip › Figure S7/hair bulb/hair bulb/results/BIDC VS NBIS.png]

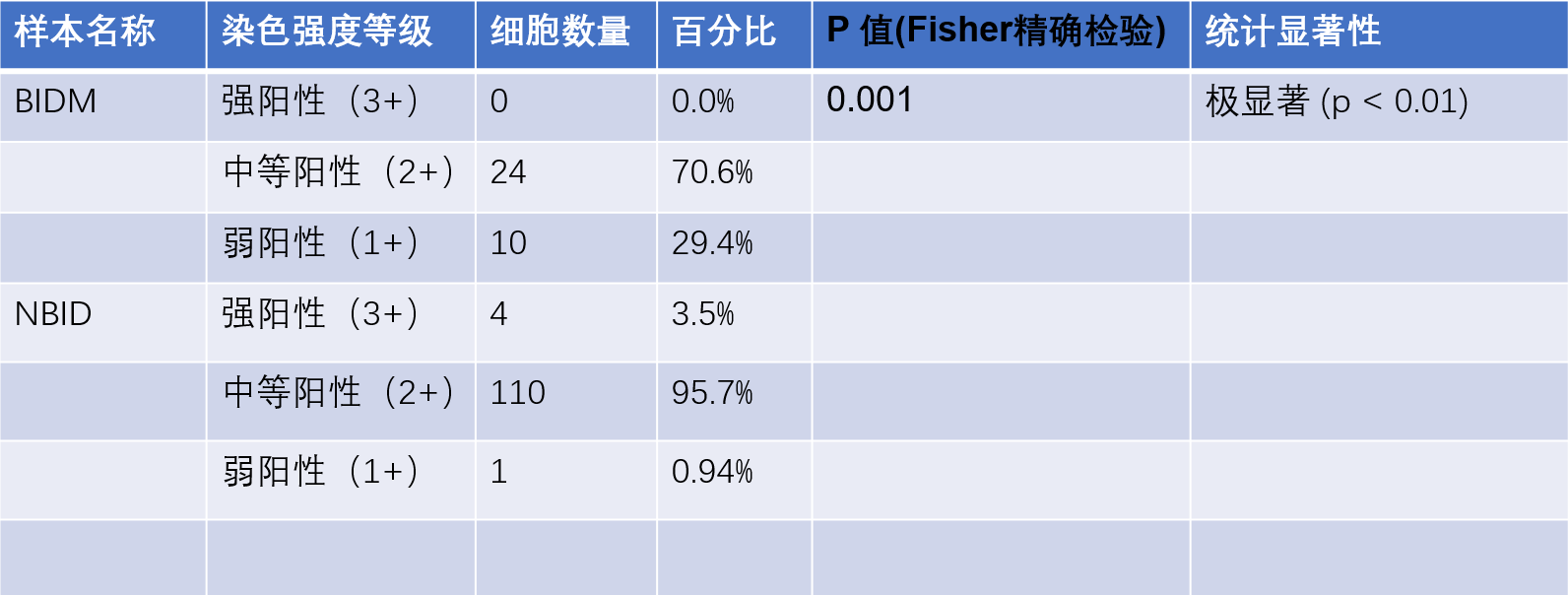

Supplement: Supplementary file 1 [file animals-16-00297-s001.zip › Figure S7/hair bulb/hair bulb/results/BIDM VS NBID.png]

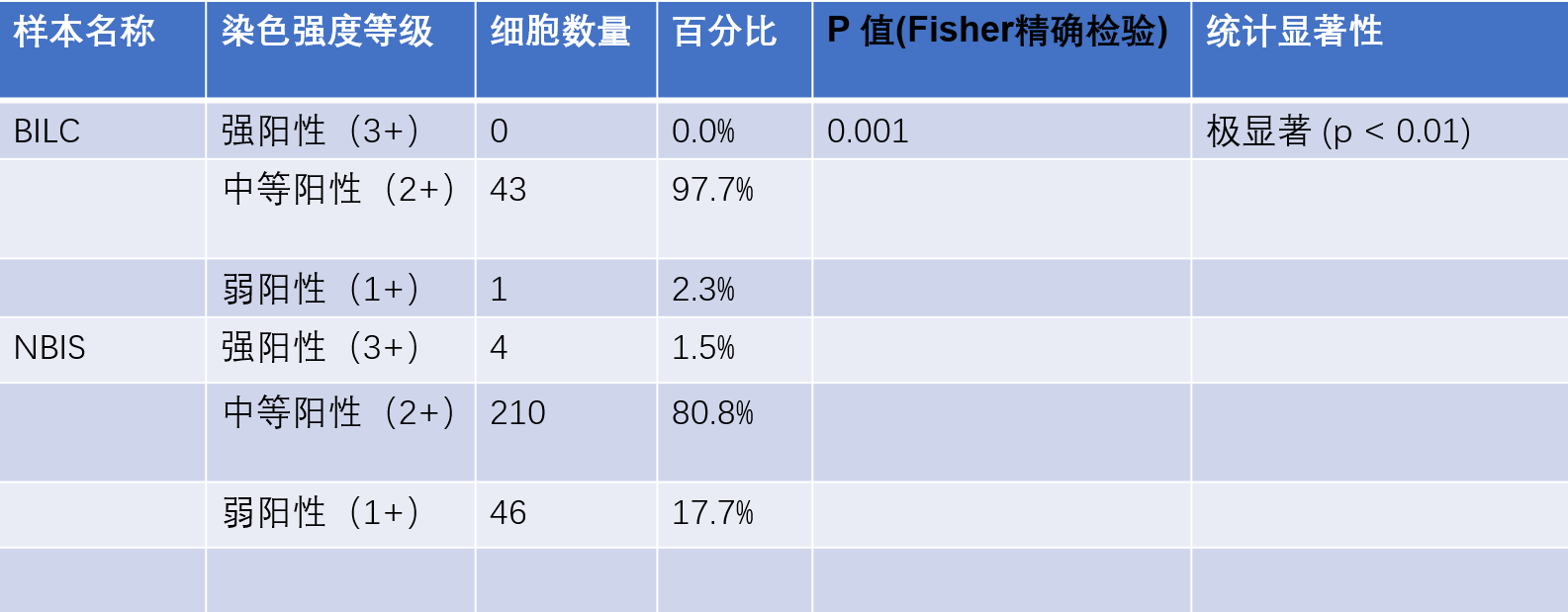

Supplement: Supplementary file 1 [file animals-16-00297-s001.zip › Figure S7/hair bulb/hair bulb/results/BILC VS NBIS.png]

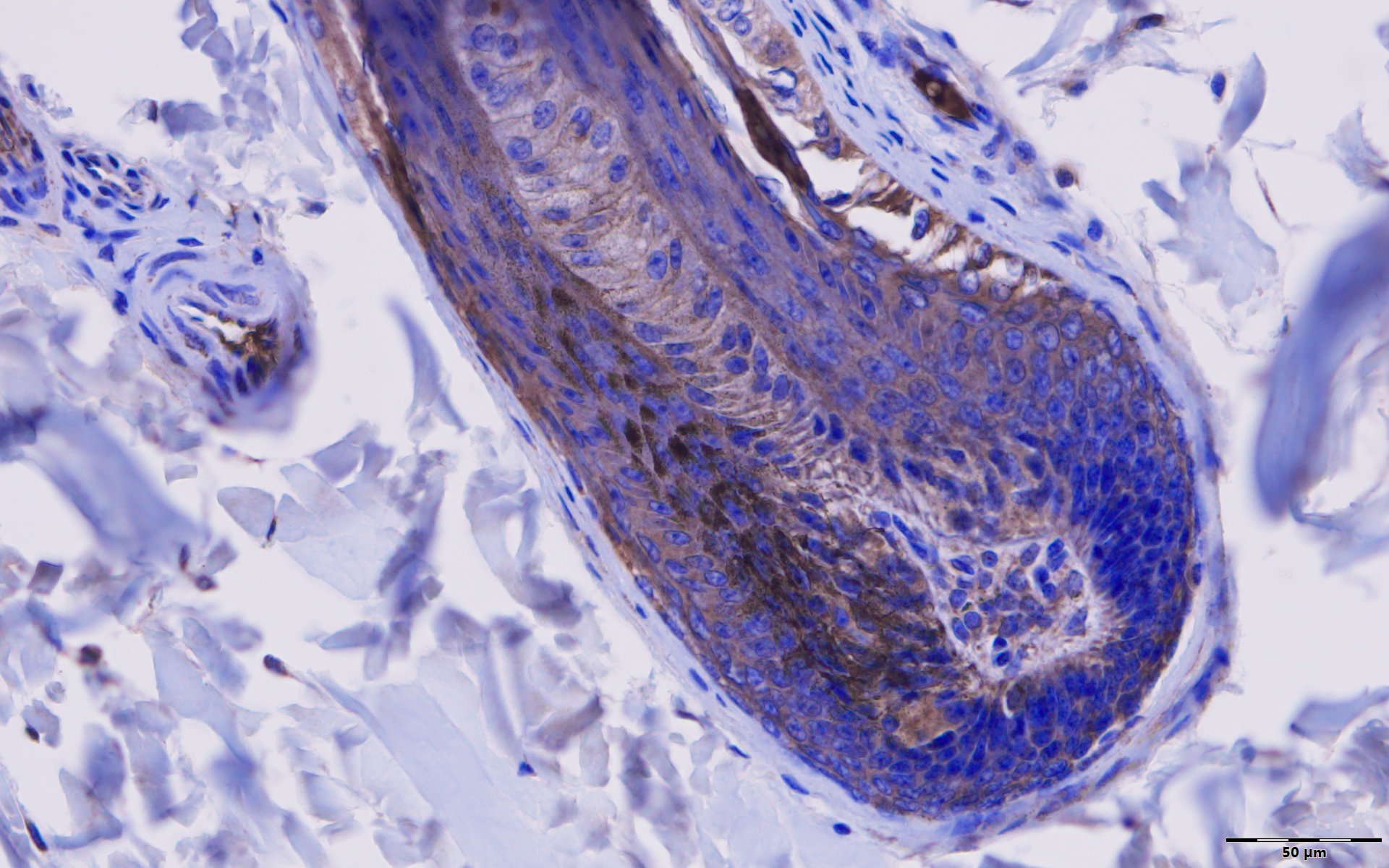

Supplement: Supplementary file 1 [file animals-16-00297-s001.zip › Figure S7/hair bulb/YBC.jpg]

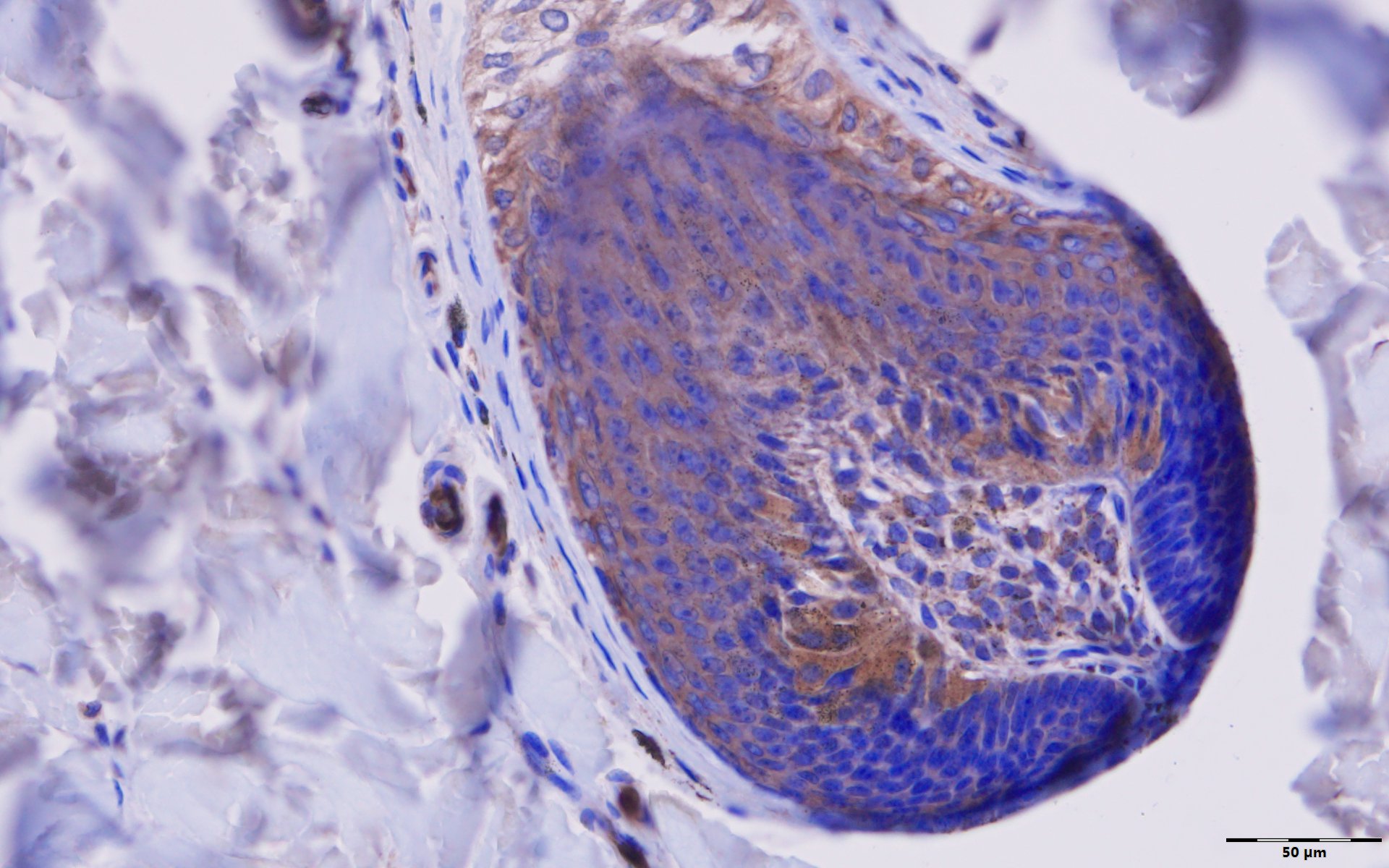

Supplement: Supplementary file 1 [file animals-16-00297-s001.zip › Figure S7/hair bulb/YBD.jpg]

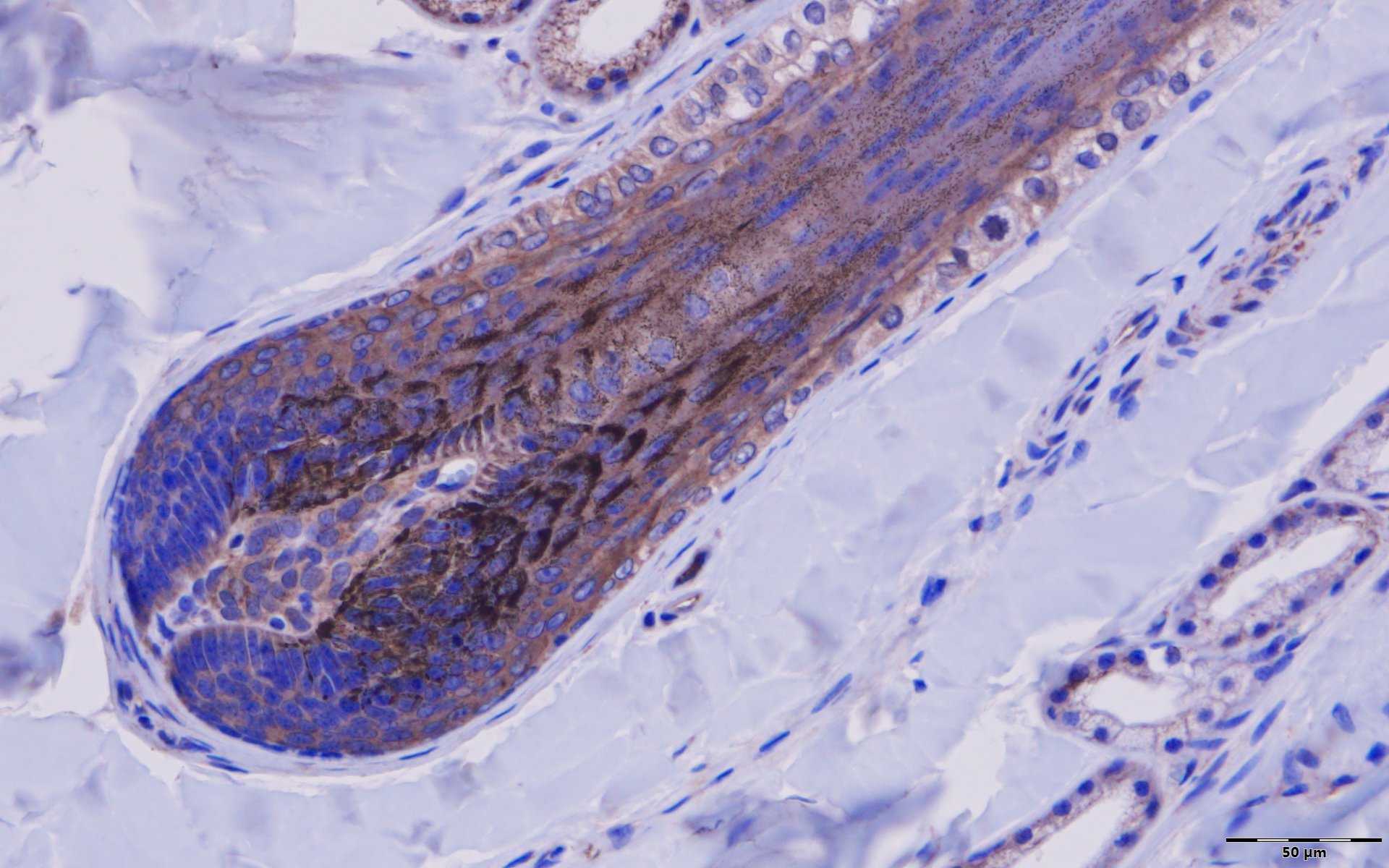

Supplement: Supplementary file 1 [file animals-16-00297-s001.zip › Figure S7/hair bulb/YBDC.jpg]

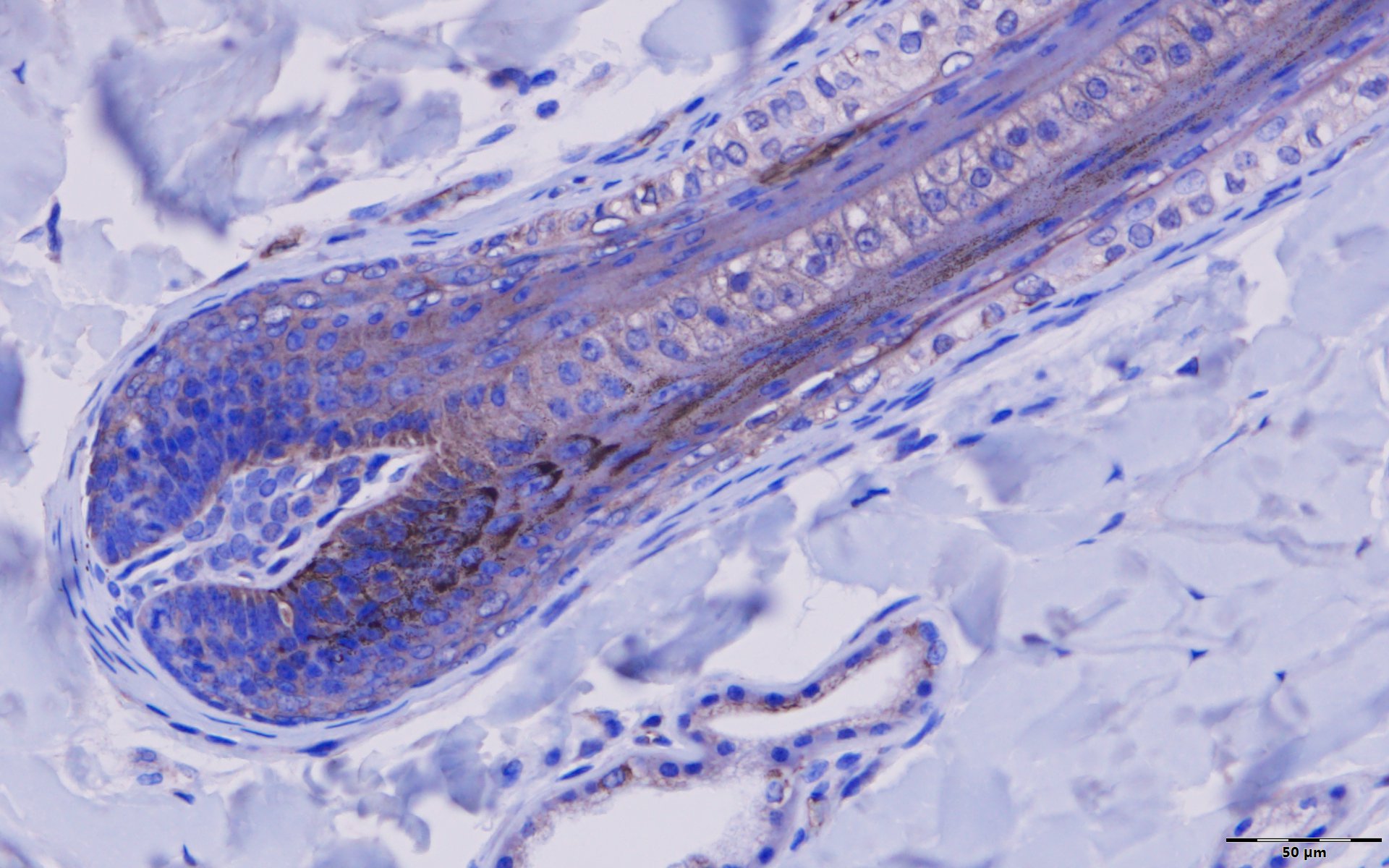

Supplement: Supplementary file 1 [file animals-16-00297-s001.zip › Figure S7/hair bulb/YBLC.jpg]
